# Novel role of circRSU1 in the progression of osteoarthritis by adjusting oxidative stress

**DOI:** 10.7150/thno.53307

**Published:** 2021-01-01

**Authors:** Yute Yang, Panyang Shen, Teng Yao, Jun Ma, Zizheng Chen, Jinjin Zhu, Zhe Gong, Shuying Shen, Xiangqian Fang

**Affiliations:** 1 Department of Orthopaedic Surgery, Sir Run Run Shaw Hospital, Zhejiang University School of Medicine.; 2 Key Laboratory of Musculoskeletal System Degeneration and Regeneration Translational Research of Zhejiang Province.

**Keywords:** osteoarthritis, oxidative stress, circRSU1, MAP3K8

## Abstract

Osteoarthritis (OA), characterized as an end-stage syndrome caused by risk factors accumulated with age, significantly impacts quality of life in the elderly. Circular RNAs (circRNAs) are receiving increasing attention regarding their role in OA progression and development; however, their role in the regulation of age-induced and oxidative stress-related OA remains unclear.

**Methods:** Herein, we explored oxidative stress in articular cartilage obtained from patients of different ages. The presence of circRSU1 was detected using RNA sequencing of H_2_O_2_-stimulated primary human articular chondrocytes (HCs), and validated in articular cartilage and HCs using fluorescence *in situ* hybridization (FISH) staining. miR-93-5p and mitogen-activated protein kinase kinase kinase 8 (MAP3K8) were identified as interactive circRSU1 partners based on annotation and target prediction databases, and their associations were identified through dual-luciferase reporter analysis. The effect of the circRSU1-miR-93-5p-MAP3K8 axis on HCs was confirmed using western blot, quantitative real-time PCR (qRT-PCR), enzyme-linked immunosorbent assay (ELISA), immunofluorescence, and reactive oxygen species (ROS) analyses. CircRSU1 and its mutant were ectopically expressed in mice to assess their effects in destabilization of the medial meniscus (DMM) in mice.

**Results:** We found a marked upregulation of circRSU1 in H_2_O_2_-treated HCs and OA articular cartilage from elderly individuals. circRSU1 was induced by IL-1β and H_2_O_2_ stimulation, and it subsequently regulated oxidative stress-triggered inflammation and extracellular matrix (ECM) maintenance in HCs, by modulating the MEK/ERK1/2 and NF-κB cascades. Ectopic expression of circRSU1 in mouse joints promoted the production of ROS and loss of ECM, which was rescued by mutation of the mir-93-5p target sequence in circRSU1.

**Conclusion:** We identified a circRSU1-miR-93-5p-MAP3K8 axis that modulates the progression of OA via oxidative stress regulation, which could serve as a potential target for OA therapy.

## Introduction

Osteoarthritis (OA), the most prevalent joint condition in older adults, often leads to pain and functional limitations and eventually degrades the quality of life 1. OA currently affects more than 50 million people in the US 2. Globally, it is estimated that patients with OA account for 25% of adults living with joint disorders, making the disease the fourth leading cause of disability 3, thus placing a heavy burden on individuals, health systems, and social care systems 4-6. Contemporary evidence of OA risk factors have indicated the prominent influence of age, female sex, previous joint injury, overweight, genetic predisposition, malalignment, and abnormal joint shape 7, 8. Moreover, population-based studies have demonstrated that increasing age is the strongest risk factor for OA 9 with people over the age of 65 expected to be more vulnerable to radiographic changes in one or more joints 10-12.

The increased incidence of OA with age is a result of cumulative exposure to various risk factors and biological age-related changes in joint structures 13, leading to a common end-stage OA syndrome 14. As the only cell type in articular cartilage, articular chondrocytes are responsible for maintaining homeostasis of the extracellular matrix (ECM) components. Hence, any biological event that disrupts their well-being or phenotypic stability can trigger the onset of OA 15. Oxidative stress is one such factor that can activate pro-inflammatory pathways, contributing to the chronic degeneration of the articular chondrocyte, followed by low-grade chronic systemic inflammation of the cartilage 16. The increased oxidative stress accompanied with elevated production of reactive oxygen species (ROS) in articular chondrocytes can ultimately trigger an inflammatory response 17, cellular senescence 18, dedifferentiation 19, and apoptosis 20. Preclinical and clinical evidence has shown that age and OA are inter-related, not inter-dependent 21, 22. Furthermore, OA animals injected with oxidized fragments are more vulnerable to OA due to upregulation of the degradation enzymes, matrix metallopoptidase (MMP)3 and MMP13 23. Mechanistically, ROS, acting as second messengers, can promote the state of OA primarily by stimulating ROS/mitogen-activated protein kinase (MAPK) 24, ROS/phosphoinositide-3-kinase (PI3K)/ serine/threonine kinase (AKT) 25, and reactive nitrogen species (RNS)/tumor necrosis factor (TNF)-α/nuclear factor kappa-light-chain-enhancer of activated B cells (NF-κB) pathways 17, 26. Thus, suppression of ROS production and its related signaling pathways is proposed as crucial in preventing the pathologic progression of OA, allowing chondrocytes to remain “young”.

Circular RNAs (circRNAs) are a series of covalently closed-loop noncoding RNAs 27, 28. CircRNAs can serve as microRNA (miRNA) or protein decoys 29-31, function as scaffolds facilitating contact between proteins 32, 33, and interfere with transcription or promote alternative splicing 34, 35. The competitive RNA-RNA interactions in circRNA-miRNA-mRNA crosstalk has gradually become a research hotspot in various diseases 29, 36, 37. Increasing evidence shows that differentially expressed circRNAs influence OA pathogenesis 38-40 by sponging miRNAs target downstream OA-associated genes.

However, the role of circRNAs in age-associated and oxidative stress-induced OA remains unclear. Here, we investigated the expression of circRSU1 in H_2_O_2_-treated chondrocytes and older medial knee cartilage, and examined its contribution to chondrocyte matrix homeostasis disorder. The regulatory mechanisms employed by circRSU1 were also investigated in our study. Our findings highlight a novel role for the circRSU1-miR-93-5p-MAP3K8 axis in ROS production and subsequent OA progression, thereby suggesting potential therapeutic targets for OA.

## Methods

### RNA-seq and Data Collection

A circRNA profile was generated from an Illumina Hiseq yielding total RNA from a mixture of primary chondrocytes from five individuals treated with 500 μM of H_2_O_2_ for five days (H_2_O_2_-MIX) compared to a mixture of negative control primary chondrocytes from the same five individuals (NC-MIX) as previously reported 41. The remaining samples were used for quantitative real-time PCR (qRT-PCR) to validate the profile. A | log_2_ (fold change) | > 1 and FDR ≤ 0.05 was regarded as significantly different.

The other datasets used in our study were obtained from the following sources: Circbase (http://www.circbase.org/) for annotation of circRNA; TargetScan (http://www.targetscan.org/vert_72/), RNAhybrid 42 and RegRNA (http://regrna2.mbc.nctu.edu.tw/) for prediction of target miRNAs of circRNAs; TargetScan, RNA22 (https://cm.jefferson.edu/rna22/), and miRDB (http://mirdb.org/) for forecasting downstream mRNAs of miRNAs. The Gene Expression Omnibus (GEO, https://www.ncbi.nlm.nih.gov/geo/, *GSE 86578*, GSM2306261, GSM2306265, GSM2306269, GSM2306268, GSM2306272, and GSM2306264) was used to detect differential mRNA expression induced by pro-inflammatory cytokines. EBI (https://www.ebi.ac.uk/Tools/psa/) was employed for pairwise sequence alignment.

### Human Cartilage Collection

Human articular cartilage was collected from patients undergoing total knee replacement surgery, as approved by the Ethics Committee of Sir Run Run Shaw Hospital (Zhejiang, China) and proceeded in accordance with the approved guidelines. Written informed consent was obtained from all participants. All knee joints (n = 30) were divided into two groups according to participant age. Those from individuals between 60 and 69 years (n = 15) were defined as younger, meanwhile those from 70-85 (n = 15) year participants were defined as older. Heterogeneity analysis of confounding factors associated with the onset of OA in the two groups, such as gender and BMI 14, between the two groups was performed. WOMAC 43, Kellgren & Lawrence, and Outbridge grade 44 were performed to evaluate the degree of OA in patients.

### Animal Models

Adult male C57BL/6 mice (n = 40), aged eight weeks, were used for *in vivo* experiments. According to a previous study 45, 46, destabilization of the medial meniscus (DMM) surgery was performed to induce post-traumatic OA as the positive control. Briefly, mice (n = 10) were anesthetized, and their medial joint capsules were incised to expose the medial meniscotibial ligament (MMTL). Subsequently, the MMTL was transected with microsurgical scissors to release the ligament linked to the tibial plateau, consequently destabilizing the medial meniscus. The joint was irrigated with sterile saline and closed. A sham operation was performed in parallel by incising the medial knee joint capsule. The adeno-associated virus (AAV) of circRSU1 and its mutant were constructed and packaged by HanBio (Shanghai, China). One week after the operation, thirty sham-operated mice were randomly divided into three groups (SHAM+NC, SHAM+circRSU1, and SHAM+circRSU1 Mut; n = 10/group). A total of 10 μL (approximately 1 × 10^11^ PFU/mL) of negative control AAV, circRSU1 AAV, or circRSU1 mutant AAV was delivered intra-articularly into the knee joints. The DMM-operated mice were also injected with negative control AAV (DMM+NC). Eight weeks later, before being sacrificed, mice were evaluated for knee pain using a series of assessments 47, including a hot plate test, knee extension test, and electric shock stimulated treadmill test. Twenty-month-old C57BL/6 mice (n = 10; one per cage) were collected for aging and spontaneous OA for subsequent analysis 46. Both knee articular cartilage were harvested for histological analysis or primary chondrocyte culture. All animal experiments were performed with the approval of the Institute of Health Sciences Institutional Animal Care and Use Committee (Zhejiang, China).

### Primary Chondrocyte Culture and Treatment

Primary articular chondrocytes were isolated from human and mouse knee cartilage (HCs, MCs). Articular cartilage was extracted, shredded, and digested with 0.25% trypsin-EDTA (Sigma-Aldrich, USA) in a constant 37 °C shaker at a speed of 200 rpm for 1 h, followed by digestion with 0.2% type II collagenase (Sigma-Aldrich, USA) in a 37 °C incubator overnight. The supernatant was filtered through a 0.075 mm strainer and centrifuged to collect the cell precipitate. Cells were washed twice with PBS and cultured in DMEM supplemented with 10% fetal bovine serum (FBS; Thermo Fisher Scientific, Waltham, MA, USA) and 1% penicillin-streptomycin. The culture was maintained in an incubator at 37 °C in a humidified atmosphere containing 5% CO_2_. At 70-80% confluence, IL-1β, purchased from R&D Systems (Minnesota, USA), was added to induce inflammation, while H_2_O_2_, purchased from Millipore (Billerica, MA, USA), was added to induce production of ROS, at the indicated concentration 46.

### Immunoblotting

Western blot, immunohistochemistry (IHC), and immunofluorescence were performed as previously described 48 and the antibodies used in our study are listed in **Table S1**. All images were quantified using Image-Pro Plus 6.0 (NIH, Bethesda, MD, USA). Log_2_ (fold of change) was used to calculate the relative gray value of specific proteins normalized to β-actin or GAPDH, for western blot results, positive cell percentage for IHC analysis 49, 50, and relative fluorescence intensity for immunofluorescence analysis 51 as previously indicated. Enzyme-linked immunosorbent assay (ELISA) was used to detect and quantify pro-inflammatory factors (IL-1β, IL-6, and TNF-a) in the culture supernatant, using a kit from KeyGen (Nanjing, China), according to the manufacturer's instructions.

### Quantitative Real-time PCR

Total RNA was extracted from primary chondrocytes using RNAEX reagent (Accurate Biotechnology, Hunan, China) according to the manufacturer's instructions. Reverse transcription of mRNAs or miRNAs to cDNA was performed using total RNA, with kits from Accurate Biotechnology (Hunan, China) or Sangon (Shanghai, China), respectively. Specific circRNAs or mRNAs were quantified with SYBR® Green Premix Pro Taq HS qPCR kit (Accurate Biotechnology, Hunan, China), and normalized to *ACTB*. Specific miRNAs were quantified with the MicroRNAs qPCR kit (Sango, Shanghai, China), and normalized to *U6*. The 2^-ΔΔCt^ method was used to calculate the relative expression. All experiments were independently performed in triplicate. All primers are listed in **Table S2**.

### Measurement of Intracellular ROS Levels

Intracellular ROS levels were evaluated using a reactive oxygen species assay kit (Beyotime, Shanghai, China). Briefly, following treatment for 48 h, 1 mL of serum-free medium supplemented with 10 μM DCFH-A was added to adherent chondrocytes and incubated at 37 °C for 20 min to allow DCFH-A to penetrate the cell membrane into the cells. Oxidized by intracellular ROS, dichlorodihydrofluorescein, a fluorescent metabolite of DCFH-A, was detected and imaged using a fluorescence microscope (Eclipse E600; Nikon Corporation, Tokyo, Japan), and the images were processed with Image-Pro Plus 6.0 (NIH, Bethesda, MD, USA). The results are presented as relative fluorescence intensity.

### RNA Fluorescence *In situ* Hybridization

Cy3-labeled CircRSU1 probes and FAM-labeled miR-93-5p probes were designed and synthesized by RiboBio (Guangzhou, China). A fluorescence *in situ* hybridization (FISH) kit (RiboBio, Guangzhou, China) was used to detect the probe signals in primary chondrocytes and tissue sections according to the manufacturer's instructions. Images were acquired using a Nikon A1Si Laser Scanning Confocal Microscope (Nikon Instruments Inc., Japan), processed with Image-Pro Plus 6.0 (NIH, Bethesda, MD, USA), and reported as the relative fluorescence intensity as previously indicated 51.

### RNA Immunoprecipitation

RNA immunoprecipitation (RIP) experiments were performed using the Magna RIP RNA-Binding Protein Immunoprecipitation kit (Millipore, Billerica, MA, USA). Briefly, HEK-293T cells were transfected with the argonaute-2 (AGO2) plasmid or vector. Approximately 1×10^7^ cells were pelleted by centrifugation and resuspended in 100 μL of RIP Lysis Buffer combined with protease inhibitor cocktail and RNase inhibitors. The cell lysates were incubated with anti-AGO2 (Millipore) antibody or anti-IgG (Millipore) and rotated at 4 °C overnight. The immunoprecipitated RNA was extracted using the RNeasy MinElute Cleanup kit (Qiagen) and reverse transcribed (AGbio) after treatment with proteinase K buffer. The expression of circRSU1 was determined using qRT-PCR and is expressed as a percentage of input.

### RNA Intervention

Small interfering RNAs (siRNAs) targeting the backsplicing junction of circRSU1 (si circRSU1) or targeting the specific site of mitogen-activated protein kinase kinase kinase 8 (si MAP3K8), as well as the mimic or inhibitor of miR-93-5p (mimic miR-93-5p, inhibitor miR-93-5p) were designed and constructed by RiboBio (Guangzhou, China). Primary chondrocytes obtained from human articular cartilage were seeded into 6-well plates at a density of 2 × 10^5^ cells/well, and transfected with siRNAs or mimic/inhibitor of miR-93-5p using Lipofectamine RNAiMAX (ThermoFisher, USA) in accordance with the manufacturer's instructions.

Stable silencing or overexpression of circRSU1 and MAP3K8 (sh-circRSU1, oe-circRSU1; sh-MAP3K8, oe-MAP3K8) were achieved by lentivirus infection. The short hairpin RNA (shRNA) sequences were designed according to the siRNA sequences. Wild-type lentivirus and its mutant circRSU1 and MAP3K8 were formulated by HanBio (Shanghai, China). Polybrene was added to the target cells at a concentration of 10 μg/mL.

### RNA Pull-down Assay

The RNA pull-down assay was performed using an RNA pull-down kit (BersinBio, Guangzhou, China) according to the manufacturer's instructions. Biotinylated circRSU1 probe was designed and synthesized by RiboBio (Guangzhou, China). In brief, 1 × 10^7^ human chondrocytes were harvested, lysed, and sonicated. C-1 magnetic beads were incubated with a circRSU1 probe or oligo probe at 25 °C for 2 h to generate probe-coated beads. These probe-coated beads were incubated with the cell lysates at 4 °C overnight to pull down circRSU1. After three sequential washes with the wash buffer, RNA complexes bound to the beads, were extracted using a RNeasy Mini kit (QIAGEN), and analyzed by qRT-PCR.

### Dual-Luciferase Reporter Assay

Binding between circRNA and miRNA, miRNA, and mRNA was validated by luciferase reporter assay. Genechem (Shanghai, China) constructed the luciferase reporter plasmids for the study, in which the 3ʹUTR sequence of circRSU1 or MAP3K8 and its mutants were inserted at the XbaI restriction site, between the *Firefly*_Luciferase-*Renilla*_Luciferase vector (hFLuc-XbaL-hRLuc). For luciferase reporter analysis, HEK-293T cells were seeded into 48-well plates and cultured to 50%-70% confluence. A miRNA mimic or mimic-NC (RiboBio, Guangzhou, China) was co-transfected with the specific luciferase reporter plasmid into HEK-293T using Lipofectamine 3000 transfection reagent (ThermoFisher, USA) according to the manufacturer's instructions. Forty-eight hours after incubation, the luciferase activity was measured using a dual-luciferase reporter assay system (Promega, Madison, WI). The relative luciferase activity was determined using the value of hRLuc standardized to hFLuc.

### Histological Analysis

Human or mouse knee articular cartilage specimens were fixed in 4% paraformaldehyde and decalcified before paraffin embedding. Each paraffin-embedded cartilage sample was sectioned into 5-μm-thick slices for subsequent histological analysis. To evaluate the proteoglycan (PG) loss, safranin O/fast green staining (0.1% Safranin O, 0.01% Fast Green solution) and Alcian blue staining (0.1% Alcian blue solution) were performed as previously described 48, after deparaffinization and hydration. OARSI Grade 52, 53 and the MANKIN Scoring System 54, 55 were used to grade the severity of cartilage degeneration by two observers blinded to group-identifying information. OA severity was recorded with the maximal score of observed cartilage.

Reconstructed imaging of the knee joint from mice was performed using a high-resolution micro-CT instrument (InspeXio SMX-225 CT FPD HR; Shimadzu Co. Ltd., Kyoto, Japan) as previously described 56. Briefly, an X-ray energy of 225 kV and an isotropic voxel size of 4 µm was performed on knee articular cartilage as well as distal femur and proximal tibia. Each knee joint was reconstructed using a data analyzer (VGStudio MAX; Volume Graphics, Heidelberg, Germany). The number of abnormally proliferating osteophytes was counted.

### Statistical Analysis

Statistical analyses were performed using SPSS v22.0. The results are presented as the mean ± standard deviation (SD). Group differences were considered statistically different at *p* < 0.05 between groups.

## Results

### Older medial knee cartilage displays more severe arthritis and higher oxidative stress

**Figure 1:**
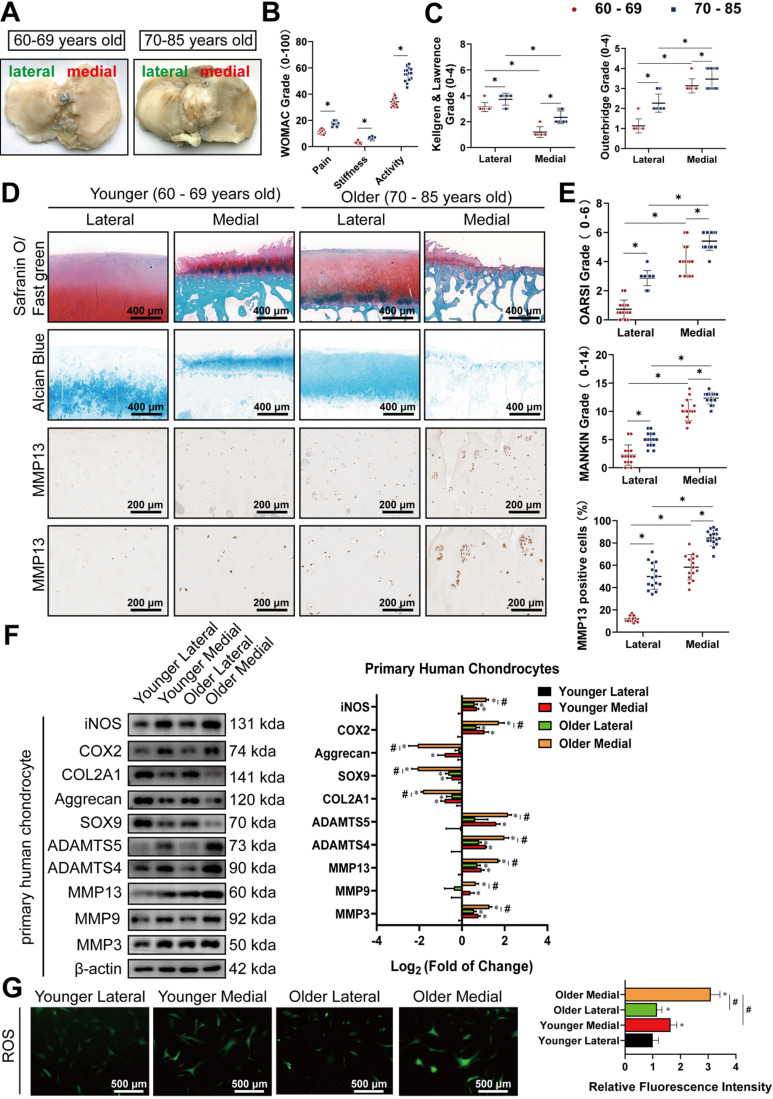
Older medial knee cartilage display more severe arthritis and higher oxidative stress. **(A)** Representative images of tibial plateau from patients of different ages after total knee arthroplasty. **(B, C)** WOMAC, Kellgren & Lawrence, and Outerbridge grade used for the evaluation of osteoarthritis symptoms in patients. (n = 15). *p < 0.05. **(D)** Representative images of Safranin O/ Fast green staining, Alcian blue staining, and immunohistochemistry (IHC) staining for MMP13 in human knee cartilage. Scale bars, 400, 200 and 100 µm.** (E)** OARSI and MANKIN grades used for assessment of histological changes of human knee cartilage. The percentage of MMP13 positive cells used for the quantification of MMP13 labeled cartilage. (n = 15). *p < 0.05. **(F) Left**, western blot analyses of primary chondrocytes from specific specimens. **Right**, quantification of western blot analyses with log_2_ (fold of change). (n = 3). *p < 0.05 compared to the chondrocytes in the Younger Lateral group. #p < 0.05. **(G) Left**, representative images of reactive oxygen species (ROS) activity of primary chondrocytes from specific specimens. Scale bars, 500 µm. **Right**, quantification of ROS activity with relative fluorescence intensity. (n = 3). *p < 0.05 compared to the Younger Lateral, #p < 0.05. Data presented as means ± standard deviation.

There were no significant differences in gender or BMI between the older and younger groups (**Table S3A, B**). Meanwhile, cartilage from the medial side of most elderly knee joints had visible subchondral bone exposure, with their surfaces rougher than those from the lateral side (**Figure 1A**). WOMAC grades showed that older patients had more severe OA symptoms (**Figure 1B**). Kellgren & Lawrence and Outbridge grades indicated that medial knee articular cartilage was more vulnerable with increased age, compared to lateral knee cartilage, based on radiological and arthroscopic observations (**Figure 1C**). Safranin O/Fast green staining, Alcian blue staining, and IHC staining for MMP13 also showed that the corruption and erosion of PG, accompanied by upregulation of MMP13, was more severe in older medial cartilage than in the corresponding lateral cartilage, or those in the younger group (**Figure 1D**). Subsequent quantification of the histomorphology changes using OARSI and MANKIN grades reflected the same conclusions (**Figure 1E**). Western blot analysis of primary chondrocytes extracted from corresponding cartilage revealed that the lateral cartilage in the older group had higher expression of degradation enzymes, including MMP3, MMP9, MMP13, a disintegrin and metalloproteinase with thrombospondin motifs (ADAMTS)4, and ADAMTS5, as well as the pro-inflammatory proteins, cyclooxygenase 2 (COX-2) and inducible nitric oxide synthase (iNOS), while exhibiting lower expression of SRY-box transcription factor 9 (SOX9), collagen type II, alpha 1 (COL2A1), and aggrecan (**Figure 1F**). ROS activities detected using the DCFH-A probe demonstrated marked increase in oxidative stress in the older group, particularly in the medial cartilage (**Figure 1G**). These findings show that disruption of medial knee cartilage and oxidative stress are more pronounced with age in arthritic patients.

### Characterization of circRSU1 in human articular chondrocytes

**Figure 2:**
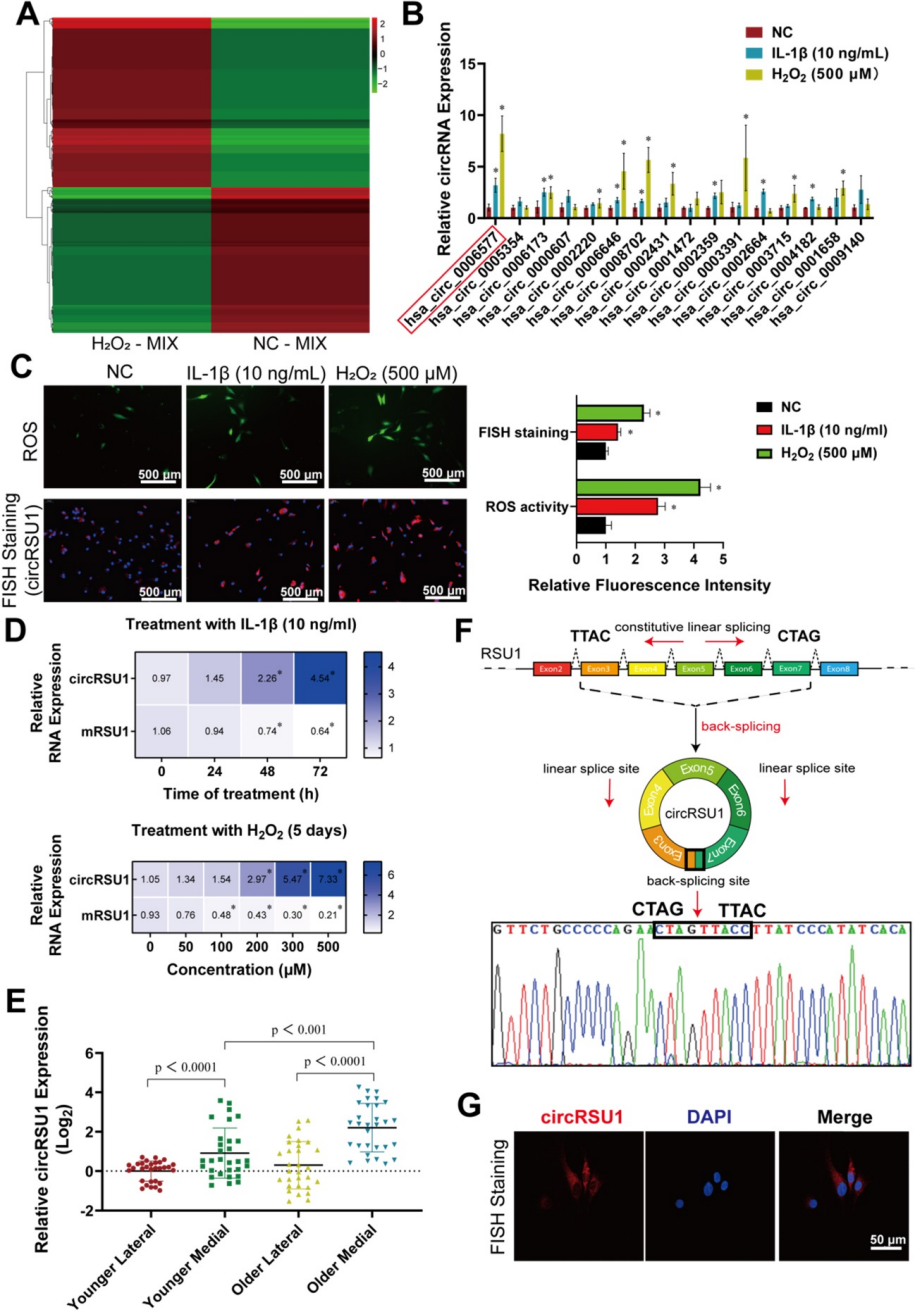
Identification of circRSU1 in human articular chondrocytes.** (A)** Heat map of all differentially expressed circRNAs, with | log_2_ (fold change) | > 1 and FDR ≤ 0.05, between a mixture of H_2_O_2_ treated chondrocytes (H_2_O_2_-MIX) and a mixture of negative control chondrocytes (NC-MIX). **(B)** Quantitative real-time PCR (qRT-PCR) quantification of the top 16 upregulated circRNAs relative expression in human articular chondrocytes (HCs) stimulated by IL-1β (10 ng/mL) and H_2_O_2_ (500 µM) for 48 h. (n = 3). *p < 0.05 compared to negative control (NC). **(C) Left**, DCFH-A-labeled reactive oxygen species (ROS) activity and circRSU1-labeled fluorescence *in situ* hybridization (FISH) staining of HCs stimulated by IL-1β and H_2_O_2_. Scale bars, 500 µm. **Right**, quantification of ROS activity and FISH staining with relative fluorescence intensity. (n = 3). *p < 0.05 compared to NC.** (D)** Blue-scale heat maps showing the RNA levels of circRSU1 and mRSU1 in HCs stimulated by IL-1β (10 ng/mL) for 0-3 days, or by gradient concentrations of H_2_O_2_ (0-500 µM) for 5 days. (n = 3). *p < 0.05 compared to NC.** (E)** qRT-PCR quantification of circRSU1 relative expression in HCs from specific sections of knee cartilage. (n = 10). **(F)** Schematic of the annotated exon formation of circRSU1; sanger sequencing of PCR production using the indicated divergent flanking primers. **(G)** Representative images of circRSU1 FISH with junction-specific probe in HCs. Scale bars, 50 µm. Data presented as means ± standard deviation.

Given that the degree of oxidative stress is consistent with the severity of arthritis, we sequenced the total RNA from H_2_O_2_-MIX compared with NC-MIX using Illumina Hiseq. A total of 27,318 circRNAs were identified by RNA-seq, most of which originated from protein-encoding exons, whereas others consisted of introns, or intergenic region (**Figure S1A**). The length of the identified circRNAs varied from 138 to 199,522 nucleotides in a skewed distribution, also indicating their complex mechanisms and functions (**Figure S1B**). Moreover, although no obvious difference was observed in the chromosomal distribution of circRNAs, total circRNA expression in the H_2_O_2_-MIX was greater than that in the NC-MIX (**Figure S1C**). A total of 550 differentially expressed circRNAs were identified by RNA-seq mapped to the reference genome (hg38, human genome), with a | log_2_ (fold change) | > 1 and FDR ≤ 0.05, 296 of which were upregulated and 254 were downregulated in the H_2_O_2_-MIX compared to NC-MIX (**Figure 2A**). qRT-PCR analysis of the remaining samples verified the RNA-seq outcome with ten upregulated and ten downregulated circRNAs (**Figure S1D**). Here, owing to the less expression difference of downregulated circRNAs (**Figure S1E**) we were more interested in the upregulated circRNAs, which may play a key role in H_2_O_2_-induced OA. There were 16 circRNAs of the top 20 upregulated circRNAs in RNA-seq matched in circBase. Thus, we perform qRT-PCR quantification to confirm the expression of these top 16 upregulated circRNAs in chondrocytes treated with IL-1β (10 ng/mL) and H_2_O_2_ (500 μM; **Figure 2B**).

Based on heat map and qRT-PCR analyses circRSU1 (hsa_circ_0006577) was selected as the most significant circRNA among other candidates (**Figure 2B**). ROS activity and relative fluorescence intensity of circRSU1 increased simultaneously in chondrocytes treated with IL-1β and H_2_O_2_ (**Figure 2C**). A chronic inflammatory environment was established by stimulating chondrocytes with IL-1β (10 ng/mL) for 0-3 days, or with gradient concentrations of H_2_O_2_ (0-500 μM) for five days 49, in which circRSU1 showed a significant dose-dependent increase, whereas linear mRSU1 showed the opposite trend (**Figure 2D**). Particularly, FISH staining of medial cartilage showed higher circRSU1 fluorescence intensity in elderly patients (**Figure S1F**). qRT-PCR analysis also confirmed the highest expression of circRSU1 in the medial cartilage of older patients (**Figure 2E**).

Thus, subsequent experiments were conducted to verify the expression and role of circRSU1. According to Circbase, circRSU1 is generated by circularization of RSU1 exons 3-7 from NM_012425, chr10 (hg19), region 16794537-16824083 (hereafter referred to as circRSU1; **Figure 2F**). The circRSU1 PCR product was sequenced to confirm the presence of the backspliced junction between the 5ʹ splice site of exon 3 and 3ʹ splice site of exon 7 (**Figure 2F**). CircRNA is reportedly more stable than mRNA due to its loop structure 27; thus, digestion with RNase R led to a significant decrease in mRSU1, but not circRSU1 (**Figure S2A**). The degradation speed of circRSU1 was much slower than that of mRSU1 when their synthesis was inhibited with actinomycin D (5 μM) for 12 h (**Figure S2B**). The lack of poly A tail implied that mRSU1 could be amplified by both oligo(dt)_18_ primers and random hexamer primers, whereas circRSU1 could only be amplified by the latter (**Figure S2C**). As indicated by a previous study 38, circRSU1 could be amplified by both convergent and divergent primers designed against its cDNA, however, could not be amplified by divergent primers designed against its genomic DNA, given its distinct backsplice (**Figure S2D**). As shown in** Figure 2G**, circRSU1 was abundant in the cytoplasm of HCs, but not in the nuclei. Taken together, these results suggest that in addition to the byproduct of splicing, the circular structure circRSU1, functionally different from the linear-structure mRSU1, is significantly upregulated in HCs from OA cartilage as well as in oxidative stress and inflammation-induced HCs.

### CircRSU1 promotes human articular chondrocyte OA

**Figure 3:**
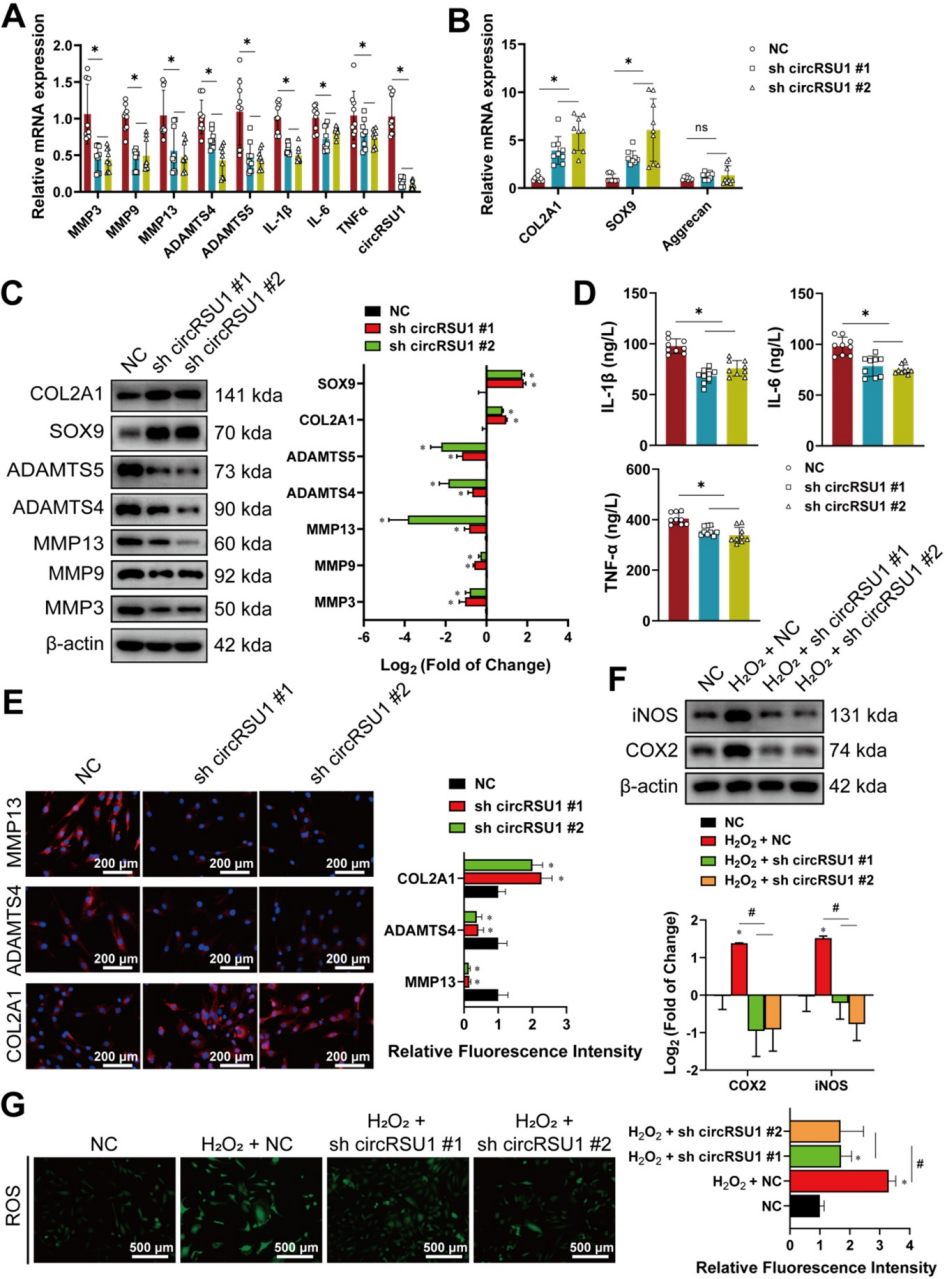
The knockdown of circRSU1 inhibits OA progression in human chondrocytes. **(A)** Quantitative real-time PCR (qRT-PCR) quantification of relative RNA levels associated with catabolic enzymes and pro-inflammatory cytokines after circRSU1 knockdown. (n = 3). *p < 0.05. **(B)** qRT-PCR quantification of relative mRNA levels associated with synthetase and proteoglycans after circRSU1 knockdown. (n = 3). *p<0.05.** (C) Left**, western blot analyses of extracellular matrix (ECM) associated protein after circRSU1 knockdown. **Right**, quantification of western blot analyses with log_2_ (fold of change). (n = 3). *p < 0.05. **(D)** Enzyme-linked immunosorbent assay (ELISA) analyses of IL-1β, IL-6 and TNF-α expression. (n = 3). *p < 0.05.** (E) Left**, representative images of MMP13, ADAMTS4 and COL2A1 labeled immunofluorescence after circRSU1 knockdown. Scale bars, 200 µm. **Right**, quantification of immunofluorescence with relative fluorescence intensity. (n = 3) *p < 0.05.** (F) Upper**, western blot analyses of reactive oxygen species (ROS) associated pro-inflammatory protein after circRSU1 knockdown, with or without H_2_O_2_ (500 µM) stimulation. **Lower**, quantification of western blot analyses with log_2_ (fold of change). (n = 3). *p < 0.05.** (G) Left**, representative images of ROS activity detected by DCFH-A probe after circRSU1 knockdown, with or without H_2_O_2_ stimulation. **Right**, quantification of ROS activity with relative fluorescence intensity. (n = 3). *p < 0.05. Data presented as means ± standard deviation.

To further study the biological function of circRSU1 in HCs, three siRNAs targeting the backspliced junction of circRSU1 were tested. Given that circRSU1-directed siRNA #1 and #2 displayed the most significant reduction in circRSU1 and did not influence the expression of linear mRSU1(**Figure S2E**), si circRSU1 #1, and si circRSU1 #2 were selected and designed as shRNAs (sh circRSU1 #1, sh circRSU1 #2) for subsequent experiments. The knockdown of circRSU1 using shRNA lentivirus in stably-transfected HCs resulted in obvious reduction of circRSU1, followed by significantly reduced mRNA levels of degradation enzymes (MMP3, MMP9, MMP13, ADAMTS4, and ADAMTS5) as well as pro-inflammatory factors (IL-1β, IL-6, and TNF-α) (**Figure 3A**), however, increased the mRNA levels of COL2A1 and SOX9 (**Figure 3B**). The results of western blot and ELISA analysis were consistent with the qRT-PCR analysis (**Figure 3C, D**). Immunofluorescence experiments further revealed that OA progression, as assessed primarily by MMP13, ADAMTS4, and COL2A1, was inhibited in HCs following circRSU1 shRNA lentivirus infection (**Figure 3E**). We found that induction of COX-2 and iNOS expression (**Figure 3F**) corresponded to the changes in ROS activities, which were stimulated by H_2_O_2_ and inhibited upon silencing of circRSU1 (**Figure 3G**).

**Figure 4:**
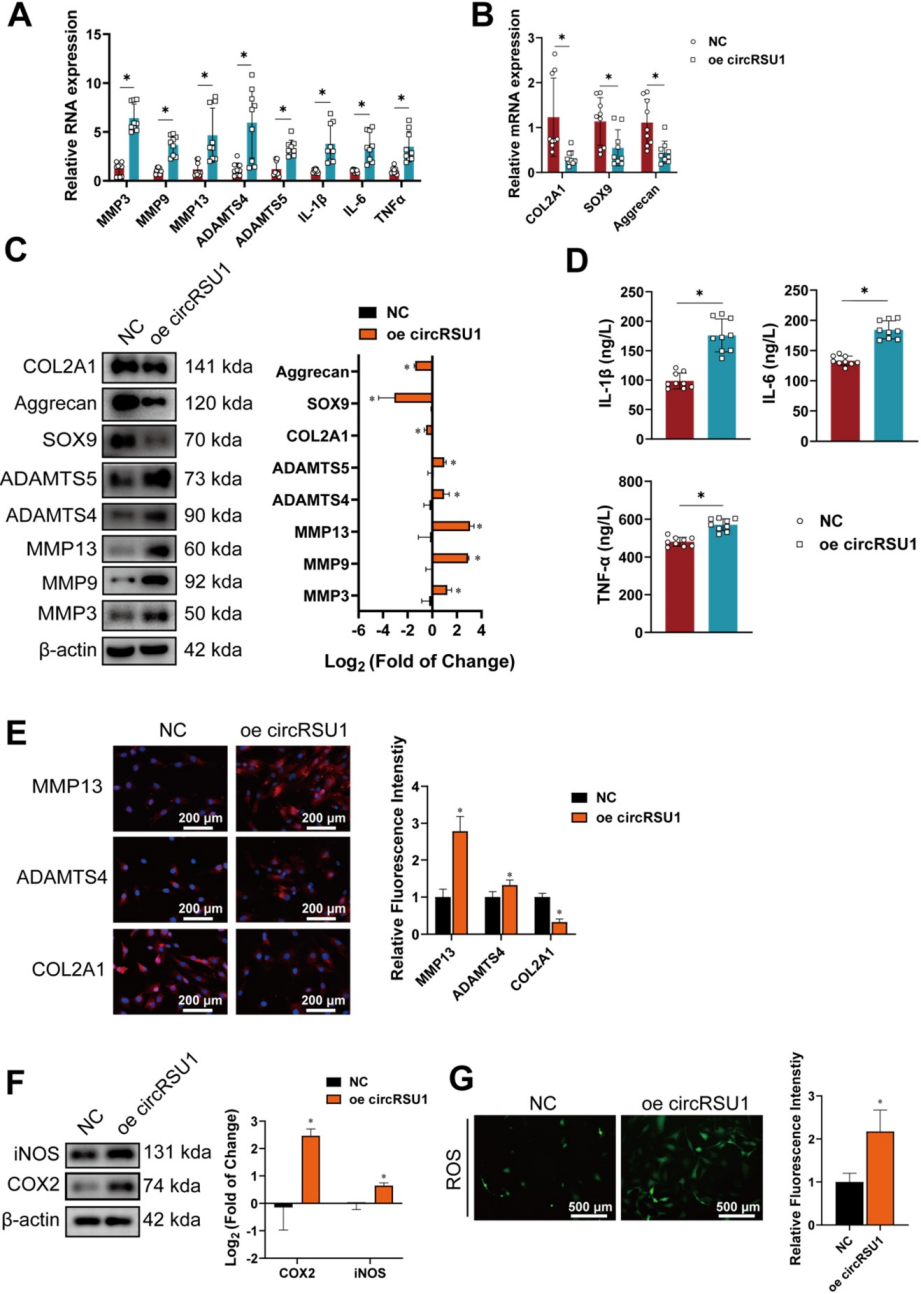
The overexpression of circRSU1 promotes the progression of osteoarthritis in human chondrocytes. **(A)** Quantitative real-time PCR (qRT-PCR) quantification of relative RNA levels associated with catabolic enzymes and proinflammatory cytokines after circRSU1 overexpression. (n = 3). *p < 0.05. **(B)** qRT-PCR quantification of relative mRNA levels associated with synthetase and proteoglycans after circRSU1 overexpression. (n = 3). *p<0.05.** (C) Left**, western blot analyses of extracellular matrix (ECM) associated proteins after circRSU1 overexpression. **Right**, quantification of western blot analyses with log_2_ (fold of change). (n = 3). *p < 0.05. **(D)** Enzyme-linked immunosorbent assay (ELISA) analyses of IL-1β, IL-6 and TNF-α expression. (n = 3). *p < 0.05.** (E) Left**, representative images of MMP13, ADAMTS4 and COL2A1 labeled immunofluorescence after circRSU1 overexpression. Scale bars, 200 µm. **Right**, quantification of immunofluorescence with relative fluorescence intensity. (n = 3). *p < 0.05.** (F) Left**, western blot analyses of reactive oxygen species (ROS)-associated proinflammatory proteins after circRSU1 overexpression. **Right**, quantification of western blot analyses with log_2_ (fold of change). (n = 3). *p < 0.05.** (G) Left**, representative images of ROS activity detected by DCFH-A probe after circRSU1 overexpression. **Right**, quantification of ROS activity with relative fluorescence intensity. (n = 3). *p < 0.05. Data presented as means ± standard deviation.

Subsequently, we studied the effect of stably overexpressing circRSU1 (oe-circRSU1) in HCs. Following infection with circRSU1-expressing lentivirus, the RNA levels of circRSU1 increased nearly hundredfold, whereas linear mRSU1 levels remained constant (**Figure S2F**). qRT-PCR revealed increases in degradative enzymes (MMP3, MMP9, MMP13, ADAMTS4, and ADAMTS5) as well as pro-inflammatory factors (IL-1β, IL-6, TNF-α) in HCs overexpressing circRSU1 (**Figure 4A**); mRNA levels of COL2A1, SOX9, and aggrecan were obviously inhibited (**Figure 4B**). Western blot and ELISA analysis also further proved the disruptive and pro-inflammatory effects of circRSU1 overexpression on HCs (**Figure 4C, D**). Relative immunofluorescence intensities of important OA markers (MMP13, ADAMTS4, COL2A1) were consistent with the above results (**Figure 4E**). Following circRSU1 overexpression, the expression of COX2 and iNOS was upregulated (**Figure 4F**). Increased oxidative stress in HCs was also detected by the DCFH-A probe (**Figure 4G**). Collectively, these data indicate that circRSU1 plays a key role in the progression of OA and production of ROS, and its effect could be reversed by circRSU1 silencing.

### CircRSU1 functions as a competing endogenous RNA for miR-93-5p

**Figure 5:**
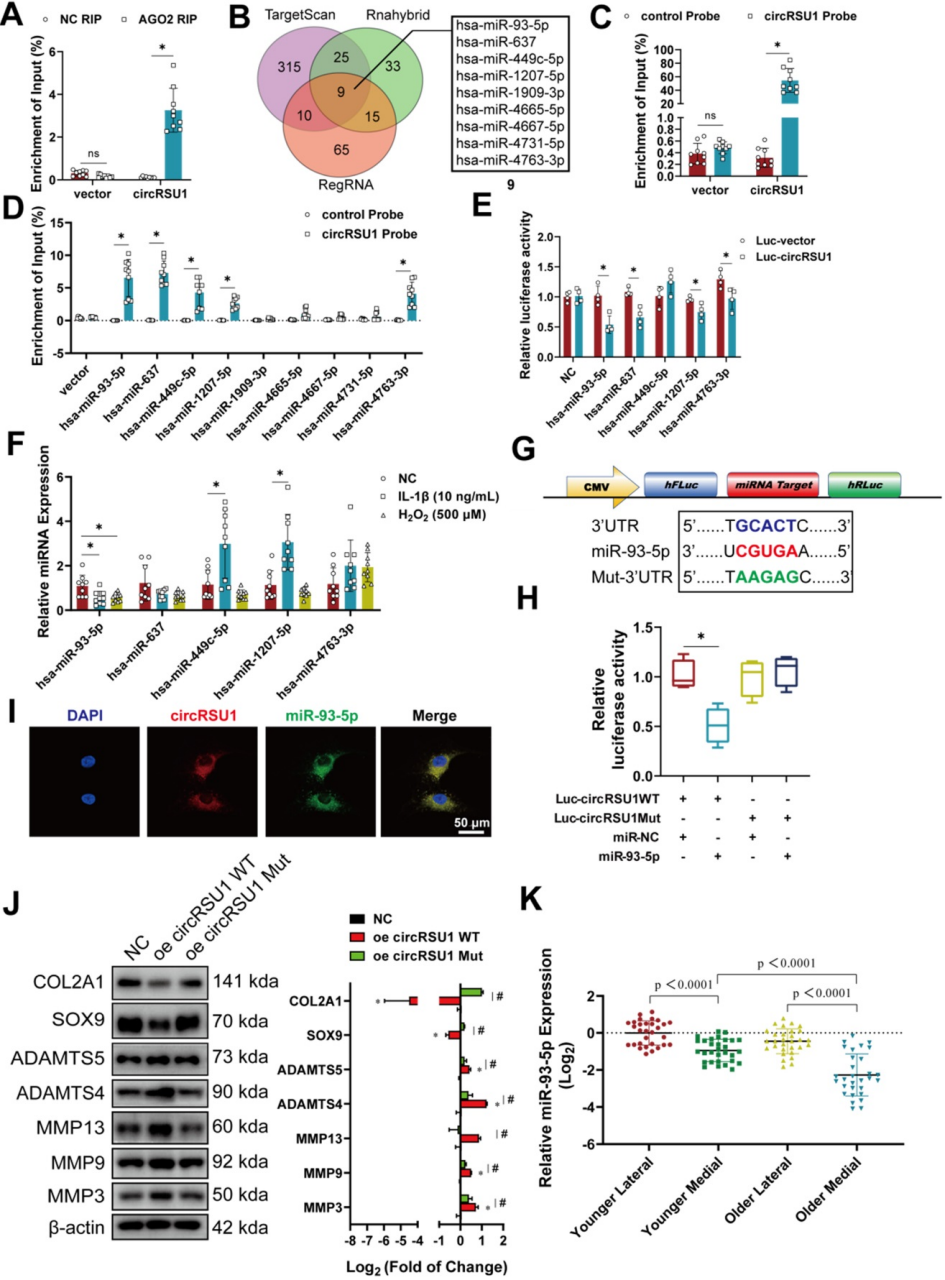
CircRSU1 functions as a competing endogenous RNA for miR-93-5p. **(A)** Quantitative real-time PCR (qRT-PCR) quantification of argonaute-2 (AGO2)-bound circRSU1, immunoprecipitated (IP)/ input values normalized to negative control (NC). (n = 3). *p < 0.05. **(B)** Venn plot showing forecast downstream miRNAs of circRSU1 converged by simultaneous analyses and overlapping of the TargetScan, Rnahybrid and RegRNA databases. **(C)** qRT-PCR quantification of the efficiency of the circRSU1 probe for RNA-pull down analysis. IP/ input values normalized to control probe. (n = 3). *p < 0.05.** (D)** qRT-PCR quantification of the circRSU1-bound miRNAs, IP/ input values normalized to control probe. (n = 3). *p < 0.05. **(E)** Relative luciferase activity of circRSU1 luciferase reporter after co-transfection with different candidate miRNAs mimics into HEK-293T cells. (n = 3). *p < 0.05.** (F)** qRT-PCR quantification of the candidate miRNAs relative expression induced by IL-1β (10 ng/mL) and H_2_O_2_ (500 µM) for 48 h. (n = 3). *p < 0.05. **(G)** Schematic of dual-luciferase reporters (hFLuc-XbaL-hRLuc), carrying wild-type or mutant circRSU1 sequence in the XbaL region. **(H)** Relative luciferase activity after co-transfection of dual-luciferase reporters (Luc-circRSU1 WT or Luc-circRSU1 Mut) with miR-93-5p mimic or its control mimic into HEK-293T cells. (n = 3). *p < 0.05. **(I)** Representative images of circRSU1 (red) and miR-93-5p (green) labeled fluorescence *in situ* hybridization (FISH) staining. Scale bars, 50 µm. **(J) Left**, western blot analyses of extracellular matrix (ECM) associated proteins after the overexpression of circRSU1 or its mutant (mutant in miR-93-5p target sequence). **Right**, quantification of western blot analyses with log_2_ (fold of change). (n = 3). *p < 0.05. **(K)** qRT-PCR quantification of miR-93-5p relative expression in primary human chondrocytes from specific parts of knee cartilage. (n = 10). *p < 0.05. Data presented as means ± standard deviation.

It has been previously proposed that circRNAs can serve as competing endogenous RNAs in the cytoplasm 37, 57, a function mediated by argonaute-2 (AGO2) 29. Given the characteristics of circRSU1, including its cytoplasmic localization in chondrocytes and enrichment by AGO2 antibody (**Figure 5A**), it was conceivable to determine whether circRSU1 could interact with miRNAs to modulate the characteristics of chondrocytes. Simultaneous assessment of TargetScan, Rnahybrid, and RegRNA databases for circRSU1 interacting partners yielded an overlap of nine candidate miRNAs (**Figure 5B**), which were identified by RNA pull-down analysis using a circRSU1 probe (**Figure 5C**). Of the nine candidate miRNAs, five miRNAs were significantly enriched in more than 5% of the input, including miR-93-5p, miR-637, miR-449c-5p miR-1207-5p, and miR-4763-3p (**Figure 5D**). Among these enriched miRNAs, miR-93-5p and miR-449c-5p exhibited better species conservation in mammals according to TargetScan databases. To validate the interaction between circRSU1 and candidate miRNAs, each of the five miRNA mimics and dual-luciferase reporter (hFLuc-circRSU1 sequence-hRLuc) were co-transfected into HEK-293T cells. As shown in** Figure 5E**, miR-93-5p, miR-637, miR-1207-5p, and miR-4763-3p mimics reduced the relative luciferase reporter activity (*Renilla* luciferase normalized to *Firefly* luciferase) compared to the negative control vector. Treatment with IL-1β or H_2_O_2_ for 48 h caused a marked decrease in miR-93-5p expression; whereas the opposite trend was observed with miR-449c-5p and miR-1207-5p, and miR-637 and miR-4763-3p remained unchanged (**Figure 5F**). Given that miR-93-5p may be the best binding candidate for circRSU1, a dual-luciferase reporter assay was subsequently performed to verify the binding site of circRSU1 on miR-93-5p. **Figure 5G** displays sequences of wild-type or mutant miR-93-5p target sites of circRSU1 according to TargetScan database, which were inserted into luciferase reporters (Luc-circRSU1 WT and Luc-circRSU1 Mut). Following co-transfection in HEK-293T cells with Luc-circRSU1 WT and mimic miR-93-5p, the relative luciferase activity was significantly reduced compared to the other combinations (**Figure 5H**), further indicating that circRSU1 provided the competing site to sponge miR-93-5p. Images obtained under a confocal microscope clearly showed the colocalization of circRSU1 (in red color) and miR-93-5p (in green color) in the cytoplasm of chondrocytes (**Figure 5I**). Overexpression of circRSU1 carrying a mutant of miR-93-5p target sequence showed recovery of overexpressed wild-type circRSU1, based on western blot analyses (**Figure 5J**). Moreover, qRT-PCR analysis of miRNA in the HCs from specific sections of the specimens also showed that the expression trend of miR-93-5p in the tissue opposed that of circRSU1 (**Figure 5K**).

**Figure 6:**
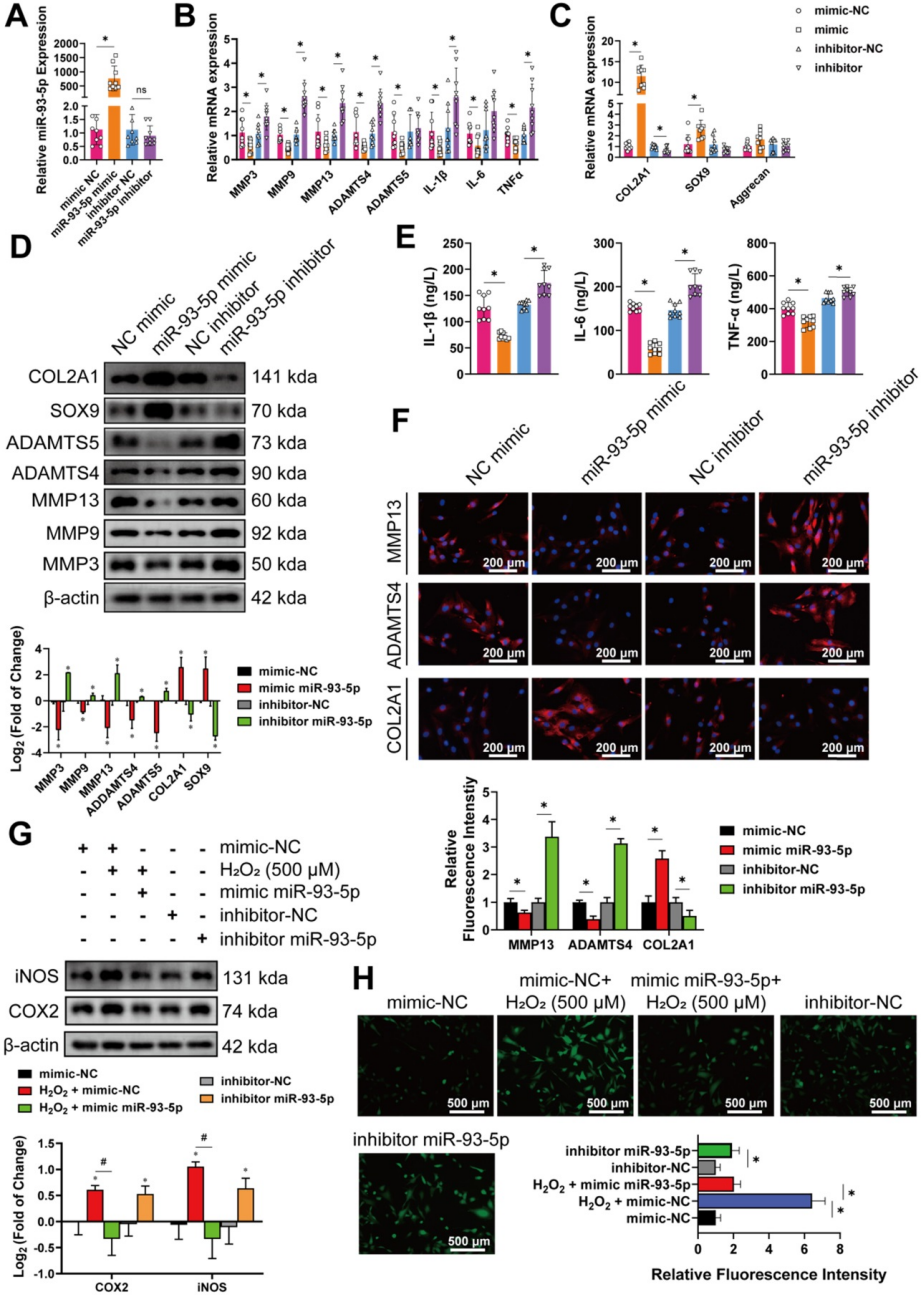
miR-93-5p promotes the progression of OA in human chondrocytes. (A) Quantitative real-time PCR (qRT-PCR) quantification of miR-93-5p relative expression regulated by its mimic or inhibitor (n=3) *p<0.05.** (B)** qRT-PCR quantification of mRNA relative levels associated with catabolic enzymes and proinflammatory cytokines after intervention for miR-93-5p (n = 3). *p < 0.05. **(C)** qRT-PCR quantification of relative mRNA levels associated with synthetase and proteoglycans after intervention for miR-93-5p. (n = 3). *p < 0.05.** (D) Upper**, western blot analyses of extracellular matrix (ECM) associated proteins after intervention for miR-93-5p. **Lower**, quantification of western blot analyses with log_2_ (fold of change) (n = 3). *p < 0.05. **(E)** Enzyme-linked immunosorbent assay (ELISA) analyses of IL-1β, IL-6 and TNF-α expression (n = 3). *p < 0.05.** (F) Upper**, representative images of MMP13, ADAMTS4 and COL2A1 labeled immunofluorescence after intervention for miR-93-5p. Scale bars, 200 µm. **Lower**, quantification of immunofluorescence with relative fluorescence intensity. (n = 3). *p < 0.05.** (G) Upper**, western blot analyses of reactive oxygen species (ROS)-associated proinflammatory proteins after intervention for miR-93-5p, with or without IL-1β (10 ng/mL) stimulation. **Lower**, quantification of western blot analyses with log_2_ (fold of change) (n = 3). *p < 0.05.** (H) Upper**, representative images of ROS activity detected by DCFH-A probe after intervention for miR-93-5p, with or without IL-1β stimulation. **Lower**, quantification of ROS activity with relative fluorescence intensity. (n = 3). *p < 0.05. Data presented as means ± standard deviation.

To explore the role of miR-93-5p in the progression of OA, miR-93-5p mimic or inhibitor was transfected into chondrocytes and compared with mimic-NC or inhibitor-NC. The expression of miR-93-5p in chondrocytes was markedly upregulated following transfection with miR-93-5p mimic, although it was modestly downregulated with miR-93-5p inhibitors (**Figure 6A**), which is likely due to the inhibitor competitively binding the miRNA without affecting its formation. Despite this, overexpression of miR-93-5p indeed reduced mRNA expression of MMP3, MMP9, MMP13, ADAMTS4, ADAMTS5, IL-1β, IL-6, and TNF-α, but increased the mRNA expression of COL2A1, and SOX9 compared to levels in control cells. Meanwhile, inhibition of miR-93-5p resulted in the opposite effect (**Figure 6B, C**). The results of western blot and ELISA corresponded to those of qRT-PCR (**Figure 6D, E**). Immunofluorescence labeling of MMP13, ADAMTS4, and COL2A1 also demonstrated similar outcomes (**Figure 6F**). To investigate the role of miR-93-5p in regulating oxidative stress in chondrocytes, western blot, and ROS detection were performed. After 24 h of IL-1 stimulation and another 24 h after transfection with the miR-93-5p mimic, the levels of COX-2 and iNOS did not increase significantly due to H_2_O_2_ stimulation (**Figure 6G**) and the activity of ROS remained protected (**Figure 6H**). However, inhibition of miR-93-5p resulted in the opposite effect (**Figure 6G, H**).

### CircRSU1-miR-93-5p-MAP3K8 axis regulates chondrocyte characteristics

**Figure 7:**
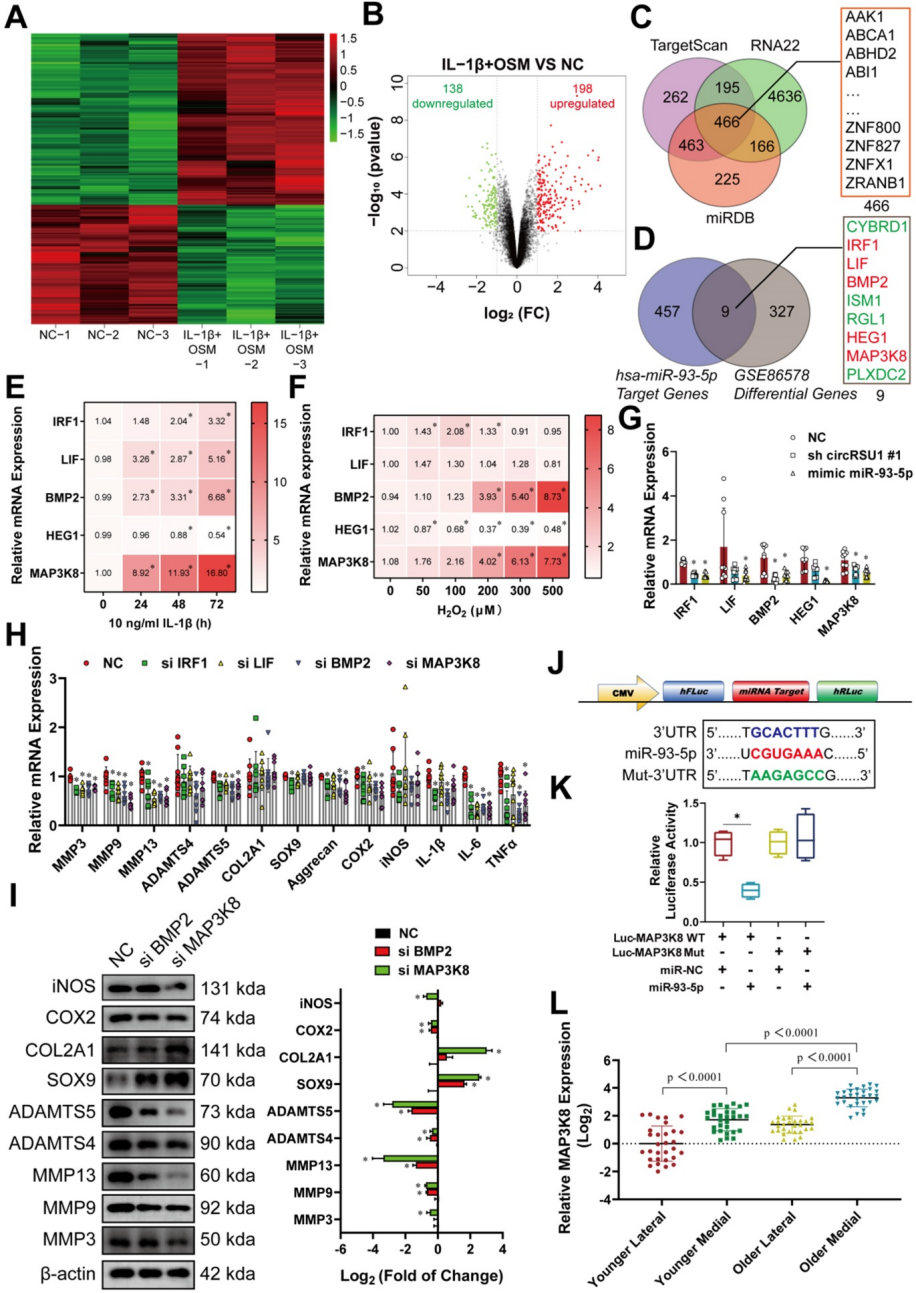
MAP3K8 is selected and confirmed as the downstream gene for circRSU1 and miR-93-5p. **(A)** Heat map abstracted from a published transcriptome profiling dataset of human chondrocytes (*GSE 86578*). **(B)** Volcano map showing 213 downregulated mRNAs and 157 upregulated mRNAs, with a |log_2_ (fold change)| ≥ 1, in IL-1β and oncostatin M (OSM) treated chondrocytes (IL-1β+OSM group) compared to the negative control (NC group).** (C)** Venn plot showing forecast downstream mRNAs of miR-93-5p converged by simultaneous analyses and overlapping of TargetScan, RNA22 and miRDB databases. **(D)** Nine mRNAs refined according to the overlapping of 466 forecast downstream mRNAs of miR-93-5p and 336 different-expressed mRNAs from *GSE86578*. The green, red, and black points represent downregulated, upregulated, and no statistically significant difference mRNAs in the IL-1β+OSM group compared with the NC group, respectively.** (E, F)** Red scale heat maps showing the RNA levels of the candidate mRNA in HCs stimulated by IL-1β (10 ng/mL) for 0-3 days, or by gradient concentrations of H_2_O_2_ (0-500 µM) for 5 days. (n = 3). *p < 0.05 compared to NC. **(G)** qRT-PCR quantification of candidate mRNA relative expression after the knockdown of circRSU1 or overexpression of miR-93-5p. (n = 3). *p < 0.05 compared to NC. **(H)** qRT-PCR quantification of mRNA relative expression associated with extracellular matrix (ECM) maintenance and proinflammatory cytokines after the knockdown of the candidate genes. (n = 3). *p < 0.05 compared to NC. **(I) Left**, western blot analyses of proteins associated with ECM maintenance and proinflammatory cytokines after the knockdown of BMP2 and MAP3K8 genes. **Right**, quantification of western blot analyses with log_2_ (fold of change). (n = 3). *p < 0.05 compared to NC. **(J)** Schematic of dual-luciferase reporters (hFLuc-XbaL-hRLuc), carrying wild-type or mutant MAP3K8 sequences in the XbaL region. **(K)** Relative luciferase activity after the co-transfection of dual-luciferase reporters (Luc-MAP3K8 WT or Luc-MAP3K8 Mut) with miR-93-5p mimic or its control mimic into HEK-293T cells. (n = 3). *p < 0.05. **(L)** qRT-PCR quantification of MAP3K8 relative expression in primary human chondrocytes from specific parts of knee cartilage. (n = 10). *p < 0.05. Data presented as means ± standard deviation.

To fully elucidate the mechanism associated with circRNA biological effects through competitive binding of miRNA, also known as a circRNA-miRNA-mRNA regulatory molecular axis, we explored potential downstream target genes. First, we employed a published transcriptome profiling dataset of human chondrocytes (*GSE 86578*), the reliability of which which was previously validated by Chan et al 58 and Macdonald et al 59. Many inflammatory mediators are reportedly involved in the pathogenesis of OA 60 thus, we used a 24-h stimulation with IL-1β (and OSM (oncostatin M) in HCs to represent the arthritis group (GSM2306268, GSM2306272, and GSM2306264), the phenotypes of which were much similar to those of arthritic chondrocytes, versus the negative control group (GSM2306261, GSM2306265, and GSM2306269). Of the 336 differentially expressed mRNAs with a | log_2_ (fold change) | ≥1, 198 were upregulated, whereas 138 were downregulated in IL-1β+ OSM-treated chondrocytes compared to control samples (**Figure 7A, B**). Simultaneous analyses and overlapping of TargetScan, RNA22, and miRDB databases converged on 466 possible downstream target genes of miR-93-5p (**Figure 7C**). Combining the two results, we identified nine target genes that may affect OA progression and could be downregulated by miR-93-5p (**Figure 7D**). Five mRNAs-interferon regulatory factor 1 (IRF1), leukemia inhibitory factor (LIF), bone morphogenetic protein 2 (BMP2), heart development protein with EGF like domains 1 (HEG1), and MAP3K8—were upregulated in IL-1β+OSM-treated chondrocytes (in red), whereas cytochrome B reductase 1 (CYBRD1), isthmin 1 (ISM1), Ral guanine nucleotide dissociation stimulator like 1 (RGL1), and plexin domain containing 2 (PLXDC2) were downregulated (in green; **Figure 7D**). Stimulation with IL-1β (10 ng/mL for 0-72 h) or H_2_O_2_ (0-500 μM for 5 days) further confirmed the role of the selected upregulated target genes. As shown on the red-scale heat map, only the expression of MAP3K8 and BMP2 mRNA increased during longer incubation with IL-1β or higher concentrations of H_2_O_2_ (**Figure 7E, F**); whereas IRF1 and LIF displayed an IL-1β-dependent increase but not H_2_O_2_ dependence, and HEG1 showed the opposite tendencies (**Figure 7E, F**). We next tested whether the candidate mRNAs were regulated by the circ-miRNA-mRNA axis using qRT-PCR analysis. Overexpression of miR-93-5p inhibited the mRNA levels of five of the candidate genes, whereas IRF1, MAP3K8, and BMP2 were downregulated by circRSU1 shRNA (**Figure 7G**). These findings were further confirmed by silencing of MAP3K8 and BMP2 expression using siRNAs (**Figure S3A**). **Figure 7H** and **Figure 7I** reveal the mRNA and protein levels of MAP3K8 and BMP2 and their ability to prevent OA progression in HCs, with MAP3K8 showing superior performance. Moreover, we used a dual-luciferase reporter, which consisted of a MAP3K8 sequence with wild-type or mutant miR-93-5p target sites, as well as *Firefly* luciferase and *Renilla* luciferase (**Figure 7J**). Co-transfection of HEK-293T cells with Luc-MAP3K8 WT reporter and mir-93-5p mimic significantly reduced the relative luciferase activity (*Renilla* luciferase/Firefly luciferase; **Figure 7K**). However, the Luc-MAP3K8 Mut reporter did not interact with miR-93-5p. Notably, medial knee articular cartilage in older patients reflected higher expression of MAP3K8 mRNA, compared to levels in the lateral sections, or in younger patients (**Figure 7L**).

**Figure 8:**
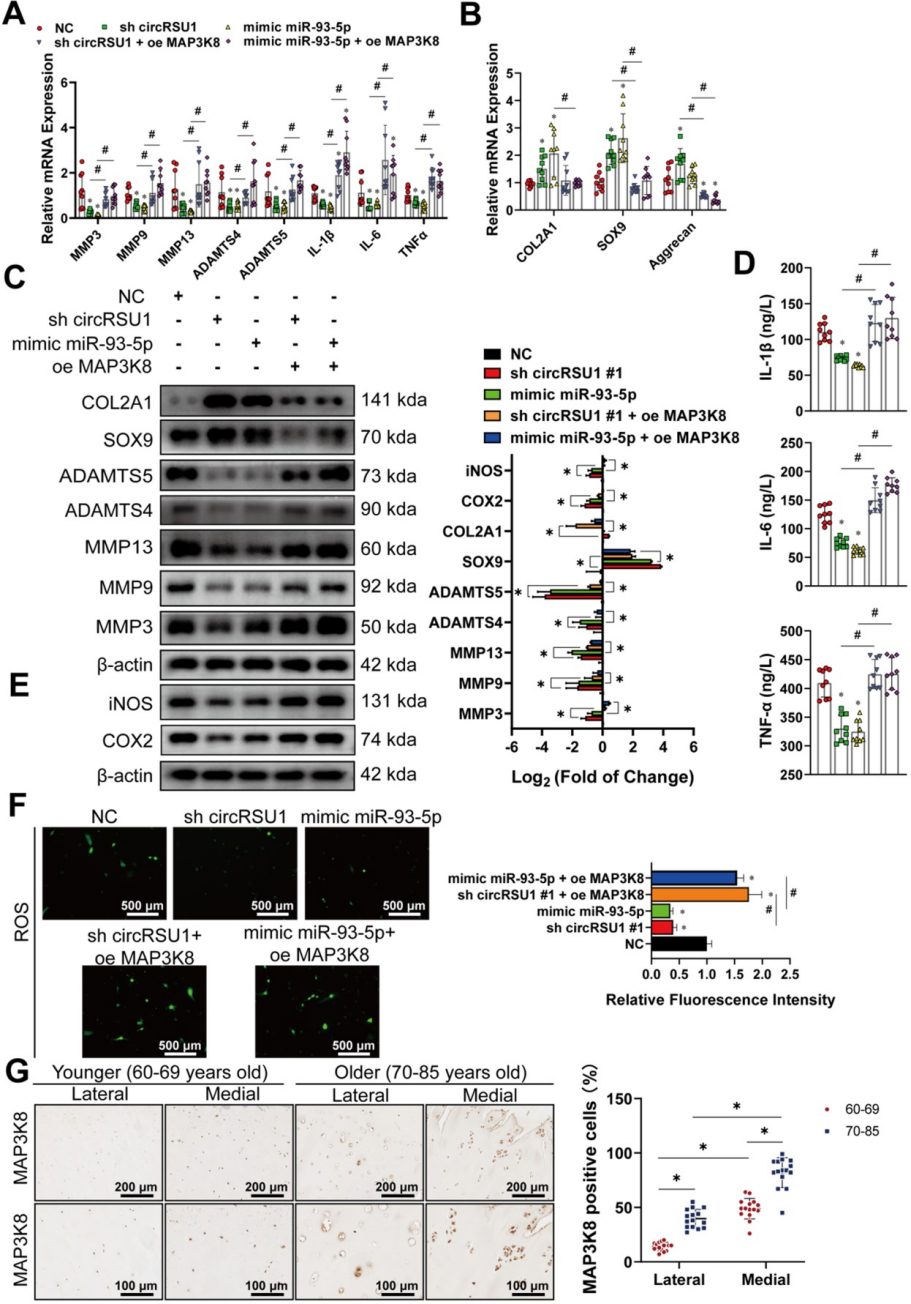
The overexpression of MAP3K8 could reverse the outcome due to transfection of circRSU1 shRNA or miR-93-5p mimic. **(A)** qRT-PCR quantification of relative mRNA expression associated with catabolic enzymes and proinflammatory cytokines after the transfection of the circRSU1 shRNA or miR-93-5p mimic, with or without overexpressed MAP3K8 (n = 3). *p < 0.05 compared to NC and #p < 0.05 **(B)** qRT-PCR quantification of relative mRNA expression related to synthetase and proteoglycans after the transfection of the circRSU1 shRNA or miR-93-5p mimic, with or without overexpressed MAP3K8 (n = 3). *p < 0.05 compared to NC and #p < 0.05. **(C) Left**, western blot analyses of extracellular matrix (ECM) associated proteins after the transfection of the circRSU1 shRNA or miR-93-5p mimic, with or without overexpressed MAP3K8. **Right**, quantification of western blot analyses with log_2_ (fold of change) (n = 3). *p < 0.05. **(D)** ELISA analysis of IL-1β, IL-6 and TNF-α expression (n = 3). *p < 0.05 compared to NC and #p < 0.05. **(E) Left**, western blot analysis of ROS-associated proinflammatory proteins expression after the transfection of the circRSU1 shRNA or miR-93-5p mimic, with or without overexpressed MAP3K8. **Right**, quantification of western blot analyses with log_2_ (fold of change) (n = 3). *p < 0.05 compared to NC.** (F) Left**, representative images of reactive oxygen species (ROS) activity detected by DCFH-A probes after the transfection of the circRSU1 shRNA or miR-93-5p mimic, with or without overexpressed MAP3K8. Scale bars, 500 µm.** Right**, quantification of ROS activity with relative fluorescence intensity (n = 3). *p < 0.05 compared to NC and #p < 0.05.** (G) Left**, representative images of MAP3K8 labeled immunohistochemistry (IHC) from specific parts of human articular cartilage. Scale bars, 200 and 100 µm. **Right**, quantification of IHC with the percentage of MAP3K8 positive cells (n=10) *p < 0.05. Data presented as means ± standard deviation.

We next assessed whether overexpression of MAP3K8 could rescue the consequences of transfection with circRSU1 shRNA or miR-93-5p mimic. The western blot analyses showed that MAP3K8 expression was efficiently downregulated or upregulated (**Figure S3B**). As OA characteristics were repressed in chondrocytes upon inhibiting circRSU1, or overexpressing miR-93-5p, upregulated MAP3K8 could reverse aberrant mRNA levels, including MMP3, MMP9, MMP13, ADAMTS4, ADAMTS5, IL-1β, IL-6, TNFα (**Figure 8A**), COL2A1, SOX9, and aggrecan (**Figure 8B**), an observation that was confirmed by western blot and ELISA analyses (**Figure 8C, D**). Moreover, a recovery was observed in the expression of COX2 and iNOS in western blot (**Figure 8E**) as well as in ROS activity, as detected by DCFH-A probe (**Figure 8F**). Immunofluorescence analysis of suppressed MAP3K8 and overexpressed MAP3K8, as shown in **Figure S3C, D**, further proved that MAP3K8 could promote the progression of arthritis in HCs. MAP3K8 labeled staining of the knee articular cartilage from younger or older specimens indicated its role in the progression of OA (**Figure 8G**). Collectively, we conclude that circRSU1 modulates the characteristics of chondrocytes by sponging miR-93-5p to regulate the expression of MAP3K8.

### The CircRSU1-miR-93-5p-MAP3K8 axis is significant and occurs via the ERK1/2 and NF-κB pathways

**Figure 9:**
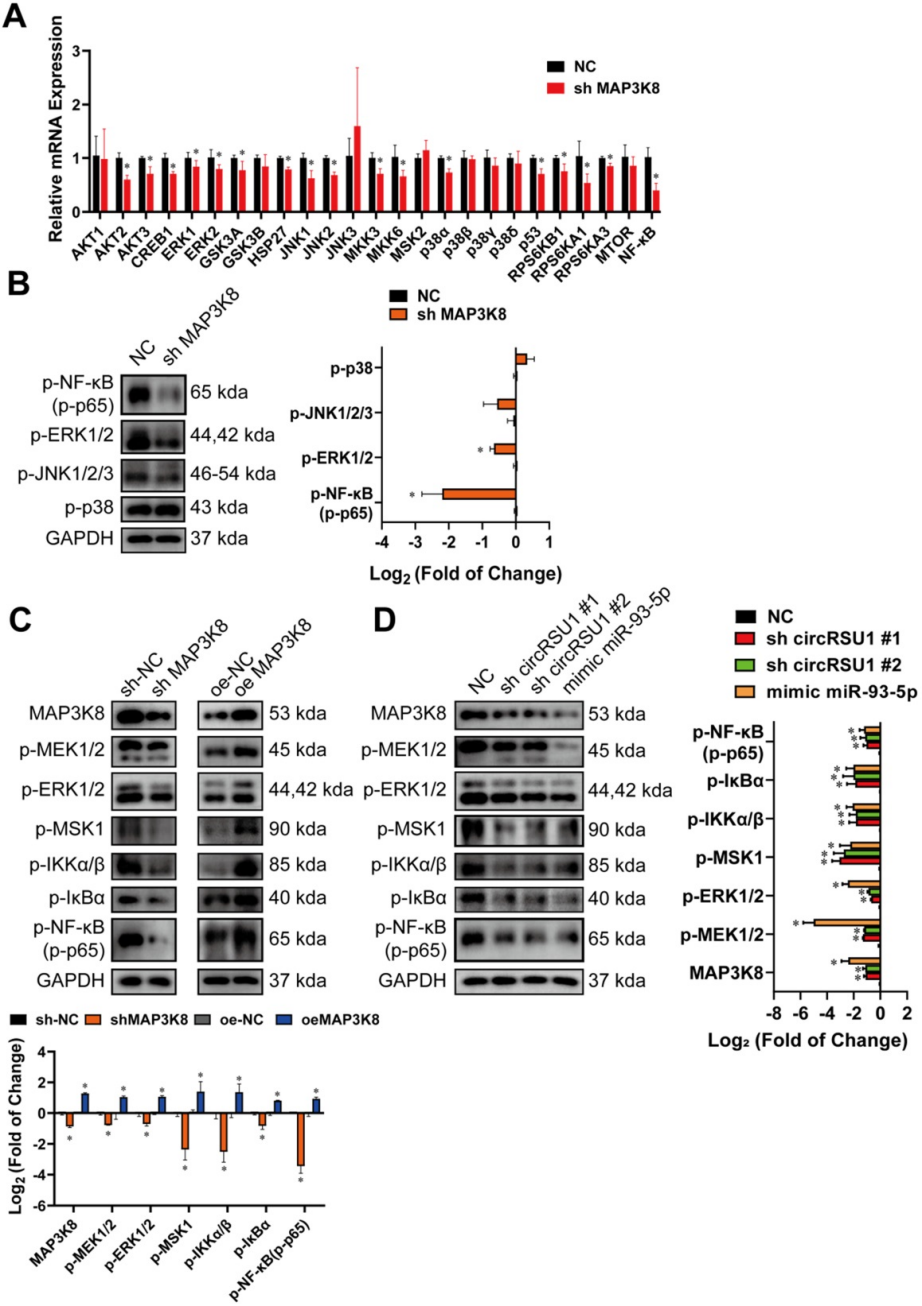
The circRSU1-miR-93-5p-MAP3K8 axis occurs via ERK1/2 and NF-κB pathways. **(A)** Quantitative real-time PCR (qRT-PCR) quantification of relative mRNA expression involved in MAPK and NF-κB pathways, after MAP3K8 silencing. (n = 3). *p < 0.05 compared to negative control (NC). **(B) Left**, western blot analyses of typical phosphorylated proteins in MAPK and NF-κB pathways, after MAP3K8 silencing. **Right**, quantification of western blot analyses with log_2_ (fold of change). (n = 3). *p < 0.05 compared to NC. **(C) Upper**, western blot analyses of marker phosphorylated proteins in MEK-ERK1/2 and NF-κB pathways, after the downregulation or upregulation of MAP3K8. **Lower**, quantification of western blot analyses with log_2_ (fold of change). (n = 3). *p < 0.05 compared to NC. **(D) Left**, western blot analyses of marker phosphorylated proteins in MEK/ERK1/2 and NF-κB pathways, after the transfection of circRSU1 shRNA or miR-93-5p mimic. **Right**, quantification of western blot analyses with log_2_ (fold of change). (n = 3). *p < 0.05 compared to NC. Data presented as means ± standard deviation.

We further investigated the pathways involved in the progression of OA by the circRSU1-miR-93-5p-MAP3K8 axis. Published observations 61-64 have shown that the MAPK and NF-κB pathways are responsible for degeneration of articular cartilage. MAP3K8 is known to regulate downstream signaling pathways such as MEK/ERK MAPK, mTOR, NF-κB, and p38 MAPK in many diseases, including tumorigenesis in mice 65, rabies virus infection 66 and a model of chronic myeloid leukemia 67. Thus, mRNA levels of downstream genes of MAPK and NF-κB signaling pathways were detected by qRT-PCR following MAP3K8 silencing. As shown in **Figure 9A**, mRNA levels of AKT2, AKT3, cAMP response element binding protein 1 (CREB1), extracellular signal-regulated kinase (ERK)1, ERK2, glycogen synthase kinase (GSK)3A, GSK3B, heat shock protein (HSP)27, mitogen-activated protein kinase 8 (JNK1), mitogen-activated protein kinase 9 (JNK2), mitogen-activated protein kinase kinase (MKK)3, MKK6, mitogen- and stress-activated kinase 2 (MSK2), p38α, p53, ribosomal protein S6 kinase (RPS6K)B1, RPS6KA1, RPS6KA3, and NF-κB were significantly reduced, indicating the excellent protective effect of downregulated MAP3K8 on chondrocytes.

Western blot was then performed to assess the phosphorylation levels of the marker proteins in the affected pathway. **Figure 9B** shows that upon MAP3K8 inhibition, phosphorylated ERK1/2 (p-EKR1/2) and phosphorylated NF-κB (p-NF-κB; p-p65) levels were markedly reduced, and phosphorylated JNK (p-JNK 1/2/3) levels was moderately abrogated, however, levels of phosphorylated p38 (p-p38) remained unchanged. Considering the results of qRT-PCR and western blot, we speculated that MAP3K8 prominently affects the ERK1/2 and NF-κB pathways in HCs. Therefore, we evaluated the activities of the major components of the two pathways (**Figure 9C**). In MAP3K8 shRNA-transfected HCs, we observed reduced phosphorylation of Ser217/ Ser221 of MEK1/2, which promotes the processing and activation of ERK1/2. We also observed decreased phosphorylation of ERK1/2 at Thr202/ Tyr204 as well as reduced phosphorylation of MSK1 at Thr581, modulated by ERK1/2. Further, IKKα/β phosphorylation at Ser176/Ser180 was markedly inhibited in MAP3K8 shRNA-transfected HCs, which contributed to the decreased phosphorylation of IκBα on Ser132. The activity of NF-κB was also inhibited, with reduced phosphorylation at Ser536. MAP3K8 overexpression caused the opposite effect on MEK1/2-ERK1/2 and NF‐κB cascades (**Figure 9C**). These data are consistent with promotion of OA following silencing of circRSU1 or upregulation of miR-93-5p (**Figure 9D**), indicating that the modulation of MEK1/2-ERK1/2 and NF-κB signaling by the circRSU1-miR-93-5p-MAP3K8 axis indeed plays a key role in HCs.

### The circRSU1-miR-93-5p-MAP3K8 axis is conserved in mice and overexpressed circRSU1 induces knee articular OA in mice

Based on published studies 68, 69, the majority of circRNAs are evolutionarily conserved and abundantly expressed across species. We obtained the sequence of hsa_circRSU1 and mmu_circRsu1 from the CircBank database to compare them using the EMBOSS Needle tool in the EBI website. Through pairwise sequence alignment, we found that 431 of the 489 nucleotides of circRSU1 were conserved between humans and mice (**Figure S4A**). Furthermore, a total of 27 nucleotides upstream and downstream of the miR-93-5p target sequences in circRSU1 were highly conserved (highlighted in **Figure S4A**). The TargetScan database also indicated the conservation of miR-93-5p and its target MAP3K8 gene in humans and mice (**Figure S4B**). Using the conservation of the circRSU1-miR-93-5p-MAP3K8 axis both in human and mice as leverage, we designed primers for mmu_circRsu1 and confirmed the presence of its backspliced junction using Sanger sequencing (**Figure S4C**). RNase R and actinomycin D treatments were also performed to validate its stable circular structure (**Figure S4D, E**), and oligo(dt)_18_ primers and random hexamer primers were used to distinguish the lack of poly A tail (**Figure S4F**). RNA FISH staining of mmu_circRsu1 also indicated the cytoplasmic localization of mmu_circRsu1 in MCs (**Figure S4G**), which was consistent with the previous results of hsa_circRSU1.

**Figure 10:**
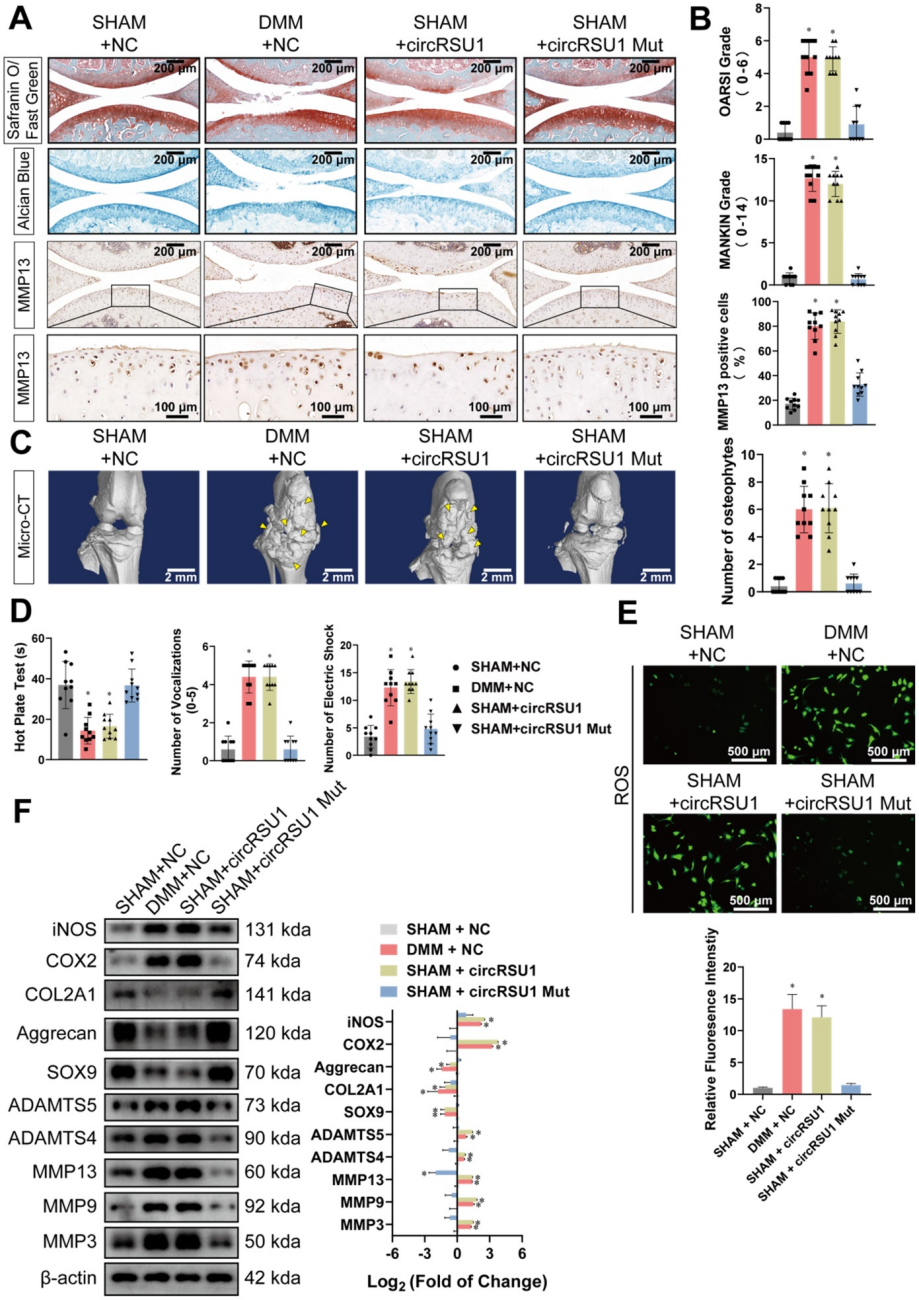
Overexpressed circRSU1 promotes the progression of OA in mice. (A) Representative images of Safranin O/ Fast green, Alcian blue, and MMP13 labeled immunohistochemistry (IHC) staining of knee cartilage from mice. Scale bars, 200 and 100 µm. **(B)** OARSI and MANKIN grade used for the assessment of histological changes of mouse knee cartilage. The percentage of MMP13 positive cells used for quantification of MMP13 labeled cartilage. (n = 10) *p < 0.05 compared to SHAM+NC group. **(C) Left**, representative three-dimensional (3D) reconstruction images of mouse knee joints showing abnormal growth of osteophytes (indicated by yellow arrow). Scale bars, 2 mm. **Right**, quantification of 3D reconstruction images by counting the number of osteophytes. (n = 10). *p < 0.05 compared to the SHAM+NC group. **(D)** Quantification of the hot plate test, vocalizations evoked by extension of the knee and knee pressure test, and electric shock stimulated treadmill test to evaluate the pain in the knee joints of mice (n = 10). *p < 0.05 compared to the SHAM+NC group. **(E) Upper**, representative images of reactive oxygen species (ROS) activity detected by DCFH-A probes in primary chondrocytes from mice knee articular cartilage. Scale bars, 500 µm. **Lower**, quantification of ROS activity with relative fluorescence intensity (n = 3). *p < 0.05 compared to the SHAM+NC group. **(F) Left**, western blot analyses of proteins associated with extracellular matrix (ECM) maintenance and proinflammatory cytokines in primary chondrocytes from mice knee articular cartilage. Right, quantification of western blot analyses with log_2_ (fold of change) (n = 3). *p < 0.05 compared to the SHAM+NC group. Data presented as means ± standard deviation. AAV: adeno-associated virus; DMM+NC: DMM-operated mice injected with negative control AAV; SHAM+NC: sham-operated mice injected with negative control AAV; SHAM+circRSU1: sham-operated mice injected with AAV carrying wild-type circRSU1; SHAM+circRSU1 Mut: sham-operated mice injected with AAV carrying mutant circRSU1.

To corroborate these findings, we further investigated the effect of circRSU1 in mice. **Figure S5A** shows similar infection efficiency of negative controlled AAV and the AAV carrying the circRSU1 gene. CircRSU1 AAV intra-articular delivery to sham-operated mice contributed to marked PG loss, the symptoms of which were similar to those of DMM-operated mice treated with vector AAV (**Figure 10A**). However, there was no significant difference in infection with circRSU1 harboring a mutant form of miR-93-5p target sequence (circRSU1 Mut AAV) compared to the sham-operated mice administered vector AAV (**Figure 10A**). Furthermore, we clearly observed upregulated expression of MMP13 in knee articular cartilage collected from circRSU1 AAV treated mice, whereas sham-operated mice injected with vector or circRSU1 Mut AAV exhibited healthier behaviors (**Figure 10A**). OARSI and MANKIN grades indicated severe pathological degeneration of cartilage in the DMM+NC group and SHAM +circRSU1 AAV group, but not in SHAM+NC group and SHAM+circRSU1 Mut AAV group (**Figure 10B**). Three-dimensional reconstruction of the knee joint from mice specimens using micro-CT scanning indicated an unbalanced bone reconstruction, manifested as increased osteophyte production (**Figure 10C**). Hot plate test, knee extension test, and electric shock stimulated treadmill test displayed greater discomfort and suffering in the knee of SHAM +circRSU1 AAV group versus SHAM+NC or SHAM+circRSU1 Mut AAV groups (**Figure 10D**). Western blot analyses and ROS activity detection in primary MCs, collected from the four groups further validated reduced degradation and oxidative stress with more synthetic ECM in SHAM+NC or SHAM+circRSU1 Mut AAV groups, compared to DMM+NC and SHAM +circRSU1 AAV groups (**Figure 10E, F**). Furthermore, using an aging mouse model to simulate the knee joints of elderly patients in accordance with published studies 46, we detected increased degradation and oxidative stress in knee articular cartilage of 20-month-old mice, compared to those of 2-month-old mice.

Based on Safranin O/Fast Green and Alcian blue staining, there was a decomposition of proteoglycan in elderly mice, accompanied by increased expression of ECM degrading enzymes, such as MMP13 (**Figure S5B**), which were quantified using OARSI and MANKIN grades, as well as percentage of MMP13 positive cells (**Figure S5C**). The expression of marker proteins involved in OA development was detected as presented in **Figure S5D.** Moreover, ROS activities were considerably promoted in MCs collected from the cartilage of elderly mice (**Figure S5E**). Consistently, there was an upregulation of mmu_circRsu1 and downregulation of mmu_miR-93-5p in articular cartilage of the elderly mice, as observed via FISH staining, compared to that in 2-month-old mice (**Figure S5F**).

## Discussion

Although immortality is an unattainable vision for everyone, maintaining a young articular cartilage in elderly patients is the ultimate goal of all orthopedic surgeons. Aging places an unprecedented burden on articular cartilage, which eventually reduces the quality of life for the elderly 70. Currently, joint replacement surgery remains the most effective treatment for patients with end-stage OA 71, compared to pharmacological treatments such as NSAIDs, which offer limited alleviation of symptoms^72^, or regenerative medicine such as mesenchymal stem cell injections, whose efficacy is unclear 73.

Upregulated intracellular ROS has been detected in cartilage obtained from older patients 74, 75, which subsequently exacerbates the pathological changes caused by aging 18, 22. Similar to previous studies, we found that the level of ROS was closely related to the degree of arthritis, showing a positive correlation. Furthermore, the medial knee articular cartilage from older patients, which bears more body weight than the lateral cartilage 76, displayed elevated ROS and more catabolic activities compared to those in younger patients. Although a few studies have focused on circRNAs in OA 38, 40, 48, 77, the relationship between circRNAs, ROS, and OA has not yet been elucidated. Considering that earlier studies have shown the role of H_2_O_2_ in the inhibition of proteoglycan biosynthesis 49, 78 and induction of ROS production, we performed RNA sequencing using H_2_O_2_-treated chondrocytes. Hsa_circ_0006577, which is backspliced from the RSU1 gene (thus named as circRSU1), was distinct among the circRNAs induced by H_2_O_2_, the upregulation of which was also confirmed in human biopsies of cartilage from older individuals. Further experiments validated the role of circRSU1 in upregulating the level of ROS in chondrocytes and its pro-inflammatory effect on chondrocytes. Silencing circRSU1 expression caused a significant reduction in iNOS and COX2 as well as catabolic enzymes, showing a wide inhibition of ECM degradation.

CircRNAs often function as a sponge for miRNAs, and circRSU1 was no exception. In our work, for the first time, we elucidated the role of the circRSU1-miR-93-5p-MAP3K8 axis in regulating the progression of OA. MAP3K8, also known as TPL-2 or COT, has been demonstrated to have an effect on obesity 79, tumor phenotype 80, 81, atherogenesis 82 and mammalian inflammation 83. Using the GEO database *(GSE 86578)*, affiliated with TargetScan, RNA22, and miRDB databases, the MAP3K8 gene was first forecasted as the target gene of miR-93-5p, and its importance was then demonstrated in age-associated OA. Previous studies have suggested that the pro-inflammatory effect of MAP3K8 gene occurs primarily through propelling MAPK and NF-κB cascades in other diseases 79-84, we further elaborated that MAP3K8 promotes the progression of OA mainly through the EKR1/2 and NF-κB pathways, which are also the downstream signaling pathways of ROS signaling as well as the typical pathways involved in OA 21. CircRSU1 and miR-93-5p also modulated phosphorylation levels of proteins in the two pathways. Thus, we could logically conclude that the circRSU1-miR-93-5p-MAP3K8 axis participated in the progression of OA principally via the MEK/ERK1/2 and NF-κB pathways.

Higher species conservation generally indicates more important biological functions. The sequence of hsa_circRSU1 was highly similar to mmu_circRSU1, with 431 of 489 bases (88.14%) conserved in both humans and mice. Furthermore, the miR-93-5p, and MAP3K8 interacting sites in circRSU1 were well conserved, thus providing the possibility of a significant effect of circRSU1 in mice. Recovery was achieved with infection of mutant circRSU1 AAV harboring mutation in miR-93-5p target sites, compared to the severe OA symptoms observed in sham-operated mice injected with circRSU1-overexpressing vectors. Given the critical role of circRSU1, we hypothesized that the miR-93-5p target sites in circRSU1 may serve as a potential target to control age-associated OA.

Nevertheless, our study could be improved by using transgenic mice with a chondrocyte-specific promoter for circRSU1, rather than using intra-articular injection to upregulate circRSU1 expression. However, clinically, the method used to transport “drugs” into articular cavities is closer to the method currently used in clinical practice, considered as a local treatment with more reliable curative effects and fewer systemic side effects. Furthermore, to limit the potential off-target risk of anti-circRSU1, there is a need to develop a safe and reliable method to weaken the circRSU1 effect by precise targeted therapy. The importance of each element in the circRSU1-miR-93-5p-MAP3K8 axis poses a variety of measures for OA therapy, including blocking the formation of circRSU1, inhibiting the activity of MAP3K8, or injecting miR-93-5p. We have suggested the pivotal role of circRSU1 and its mutant in established OA mouse models, providing a potential site for the management of OA. Thus, the development of a circRSU1-specific drug targeting the miR-93-5p site would be a more beneficial treatment, after testing its safety and efficacy. For instance, a designed antisense RNA sequence would not only recognize the backsplicing site of circRSU1, but also competitively bind to the miR-93-5p target sequence.

Another limitation lies in the connection between mRSU1 and circRSU1. A previous review summarized that competition between splicing and backsplicing could regulate the biogenesis of circRNA 85. Given the cytoplasmic localization and exonic formation of circRSU1, as well as the opposite expression trend of circRSU1 and linear mRSU1 under IL-1β and H_2_O_2_ stimulation, we hypothesized the presence of a mechanism that regulates their biogenesis. Xu et al. 86 reported that circSMARCA5 can form an R-loop at its parent gene locus, which results in transcriptional pausing of SMARCA5 and leads to the upregulated circSMARCA5 and downregulated mSMARCA5 in breast cancer. Zhang et al. 87 also reported a mechanism for the selection of RNA pairing across flanking introns or within a single individual intron that leads to competition between the back-splicing of circRNAs and the canonical splicing of linear mRNAs. These actions are performed by specific RNA binding proteins (RBPs) and therefore, the biogenesis of circRSU1 was hypothesized to be post-transcriptionally regulated by specific RBPs. However, it is still unclear if its corresponding linear mRSU1 is regulated by circRSU1 itself or specific RBPs. Interestingly, RSU1 was originally regarded as a suppressor of Ras-induced transformation 88, 89, and its effect was further validated on several kinases downstream of the *Ras* oncogene, such as inhibition of JNK and activation of ERK 90. In our work, we revealed that the circRSU1-miR-93-5p-MAP3K8 axis regulated kinase phosphorylation in the MEK/ERK1/2 pathway. Specifically, MAP3K8 reportedly has a significant effect on Ras-induced inflammation signaling 84. Therefore, further investigation is needed to explore possible feedback regulation among the networks of circRSU1, mRSU1, MAP3K8, and Ras proteins.

In summary, our study describes, for the first time, the predominant presence of circRSU1 in cartilage from age-associated OA, as well as IL-1β- and H_2_O_2_-stimulated chondrocytes. Furthermore, we reveal a circRSU1-miR-93-5p-MAP3K8 axis as pivotal to the role of circRSU1 in regulating oxidative stress and ECM homeostasis in human chondrocytes, which occurs via the MEK/ERK1/2 and NF-κB pathways. The circRSU1-centered axis also exerts a regulatory function in established OA mouse models due to its superior conservation across species, which together, presents circRSU1 as a potential target for the treatment of age-associated OA.

## Supplementary Material

Supplementary figures and tables.

## Abbreviations

OA: osteoarthritis
circRNAs: circular RNAs
MAP3K8: mitogen-activated protein kinase kinase kinase 8
HCs: human articular chondrocytes
qRT-PCR: quantitative real-time PCR
ELISA: enzyme-linked immunosorbent assay
ROS: reactive oxygen species
DMM: destabilization of the medial meniscus
ECM: extracellular matrix
MMP: matrix metallopoptidase
MAPK: mitogen-activated protein kinase
PI3K: phosphoinositide-3-kinase
AKT: serine/threonine kinase
RNS: reactive nitrogen species
TNF: tumor necrosis factor
NF-κB: nuclear factor kappa-light-chain-enhancer of activated B cells
miRNA: microRNA
H_2_O_2_-MIX: a mixture of primary chondrocytes from five individuals treated with 500 μM of H_2_O_2_ for five days
NC-MIX: a mixture of negative control primary chondrocytes from the same five individuals
MMTL: medial meniscotibial ligament
AAV: adeno-associated virus
DMM+NC: DMM-operated mice injected with negative controlled AAV
SHAM+NC: sham-operated mice injected with negative control AAV
SHAM+circRSU1: sham-operated mice injected with AAV carrying wild-type circRSU1
SHAM+circRSU1 Mut: sham-operated mice injected with AAV carrying mutant circRSU1
MCs: mouse knee articular chondrocytes
FBS: fetal bovine serum
IHC: immunohistochemistry
FISH: fluorescence *in situ* hybridization
AGO2: argonaute-2
RIP: RNA immunoprecipitation
siRNAs: small interfering RNAs
shRNA: short hairpin RNA
PG: proteoglycan
SD: standard deviation
OSM: oncostatin M
ADAMTS: a disintegrin and metalloproteinase with thrombospondin motifs
COX-2: cyclooxygenase 2
iNOS: inducible nitric oxide synthase
SOX9: SRY-box transcription factor 9
COL2A1: collagen type II, alpha 1
IRF1: interferon regulatory factor 1
LIF: leukemia inhibitory factor
BMP2: bone morphogenetic protein 2
HEG1: heart development protein with EGF like domains 1
CYBRD1: cytochrome B reductase 1
ISM1: isthmin 1
RGL1: Ral guanine nucleotide dissociation stimulator like 1
PLXDC2: plexin domain containing 2
CREB1: cAMP response element binding protein 1
ERK: extracellular signal-regulated kinase
GSK: glycogen synthase kinase
HSP: heat shock protein
JNK1: mitogen-activated protein kinase 8
JNK2: mitogen-activated protein kinase 9
MKK: mitogen-activated protein kinase kinase
MSK: mitogen- and stress-activated kinase
RPS6K: ribosomal protein S6 kinase
