# Supplementary material for: Novel role of circRSU1 in the progression of osteoarthritis by adjusting oxidative stress
Source: Theranostics. 2021 Jan 1;11(4):1877–900. doi: 10.7150/thno.53307 (PMC7778608; doi:10.7150/thno.53307)
Supplement: Supplementary file 1 — Supplementary figures and tables. [file thnov11p1877s1.pdf]

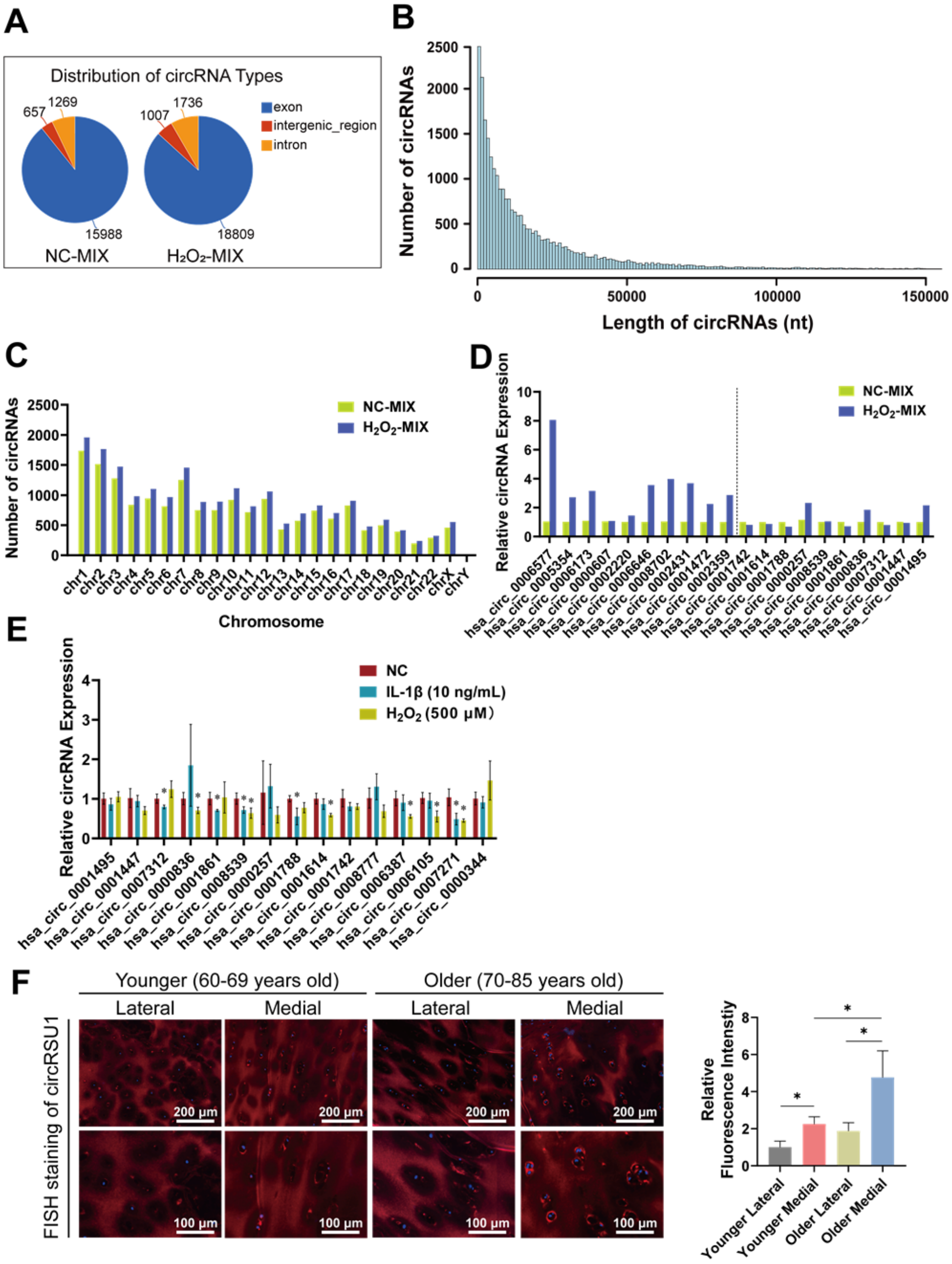

Figure S1.

Identification of a circRNA profile between a mixture of H_2O_2 treated chondrocytes (H_2O_2-MIX) and a mixture of negative control chondrocytes (NC-MIX). (**A**) Pie chart showing different genomic formation of the circRNAs in profile. (**B**) Numbers of identified circRNAs with different lengths. (**C**) Distribution of identified circRNAs in different chromosomes. (**D**) Quantitative real-time PCR (qRT-PCR) quantification of 10 upregulated and 10 downregulated circRNAs relative expression in remaining samples of H_2O_2-MIX and NC-MIX. (**E**) Quantitative real-time PCR (qRT-PCR) quantification of the top 15 downregulated circRNAs relative expression in human articular chondrocytes (HCs) stimulated by IL-1β (10 ng/mL) and H_2O_2 (500 μM) for 48 h.

(n = 3). *p < 0.05 compared to negative control (NC). (**F**) **Left**, representative images of circRSU1 fluorescence *in situ* hybridization (FISH) in specific human knee joint cartilage. Scale bars, 200 and 100 μm. **Right**, quantification of FISH with relative fluorescence intensity (n = 3). *p < 0.05. Data presented as means ± standard deviation.

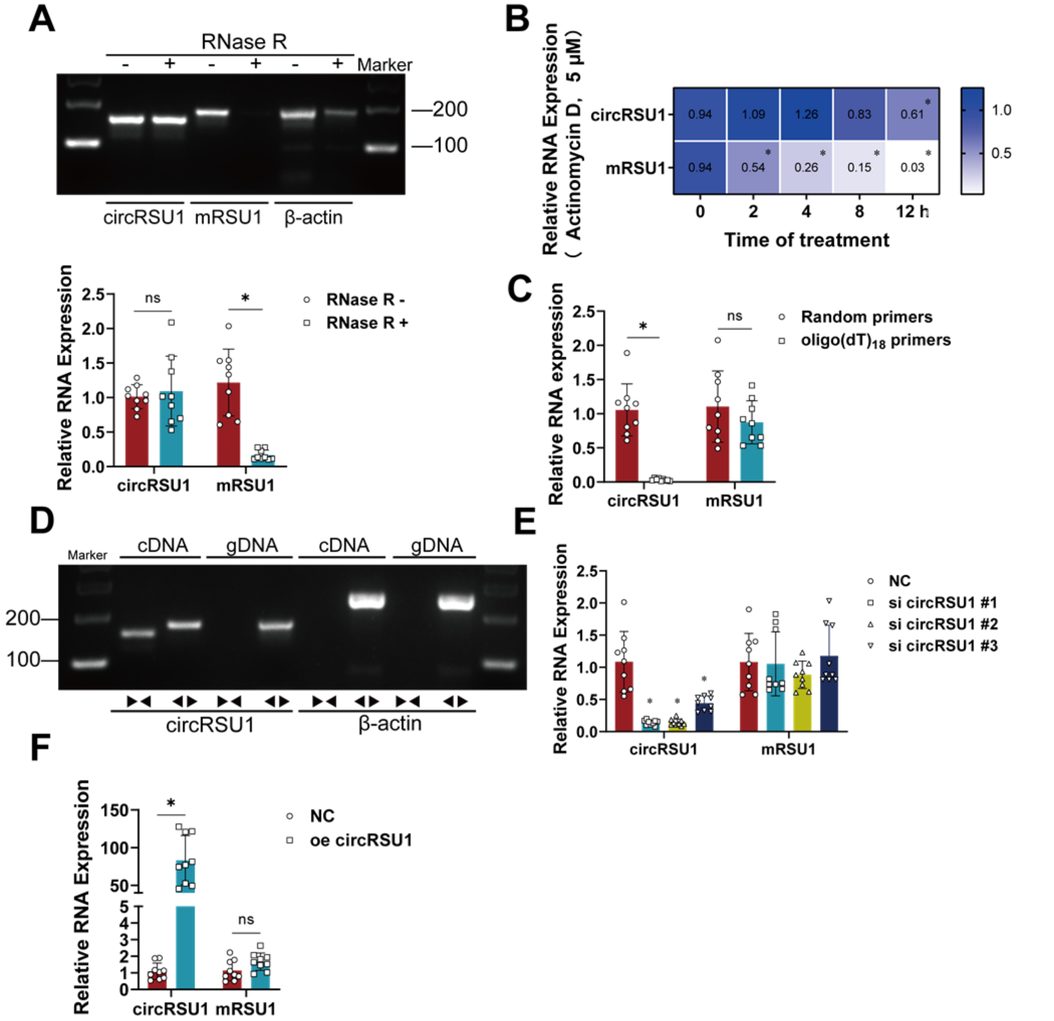

Figure S2.

Characterization of circRSU1 as a circular RNA and the efficiency of circRSU1 downregulation or upregulation. (**A**) Polymerase chain reaction (PCR) and quantitative real-time PCR (qRT-PCR) to confirm the stability of the loop structure in circRSU1 compared to linear mRSU1 after digestion with RNase R. (n = 3). *p < 0.05. (**B**) Blue scale heat map showing the stability of circRSU1 under treatment with actinomycin D (5 μM) for 12 hours. (n = 3). *p < 0.05. (**C**) qRT-PCR quantification of circRSU1 or mRSU1 relative expression using oligo(dt)$_{18}$ primers or random hexamer primers. (n = 3). *p < 0.05. (**D**) PCR using convergent and divergent primers of circRSU1 in cDNA or genomic DNA. (**E**) qRT-PCR quantification of circRSU1 and mRSU1 relative expression after knockdown with junction-specific small interfering RNAs (siRNAs) of circRSU1. (n = 3). *p < 0.05. (**F**) qRT-PCR quantification of circRSU1 and mRSU1 relative expression after infection with overexpressed circRSU1 adeno-associated virus. (n = 3). *p < 0.05. Data presented as means ± standard deviation.

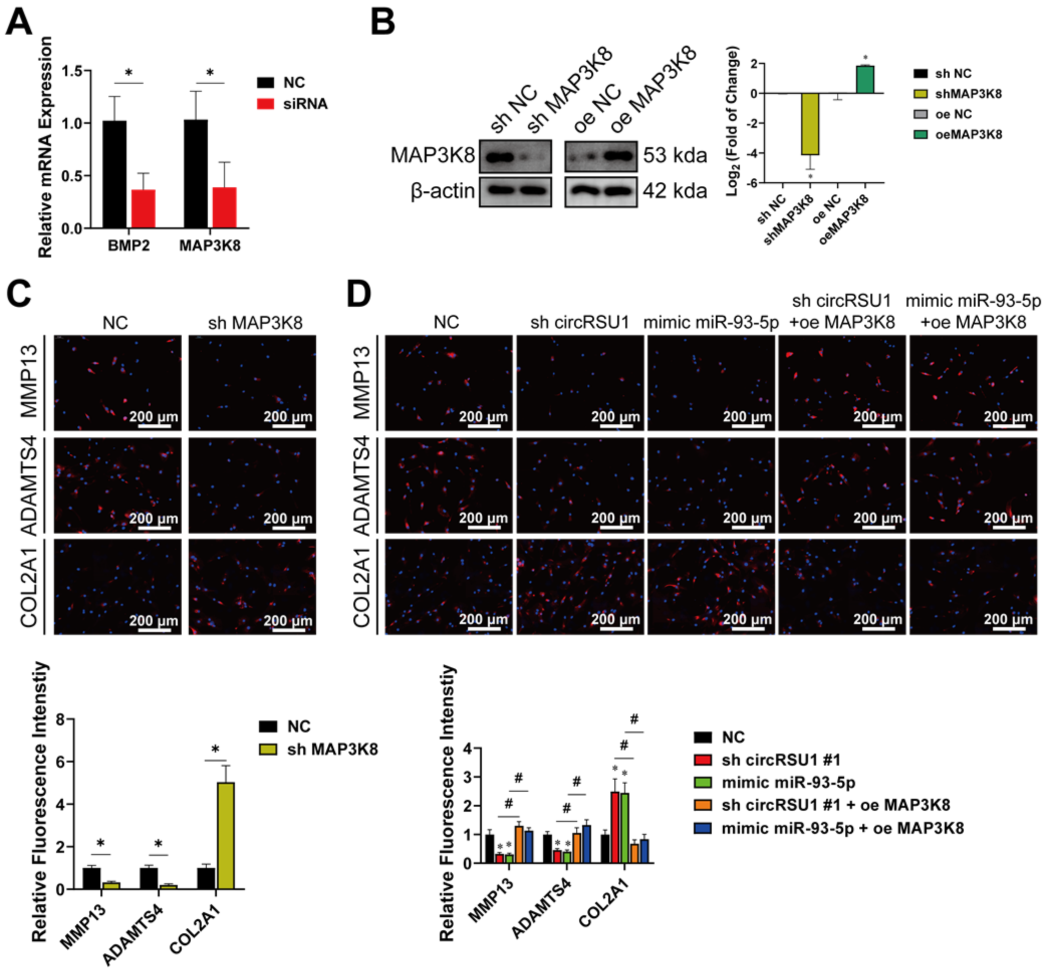

Figure S3.

(A) qRT-PCR quantification of BMP2 and MAP3K8 relative expression using corresponding small interfering RNA (siRNA). (n = 3). *$p < 0.05$. **(B) Left and middle**, Western blotting analysis of MAP3K8 protein after its downregulation or upregulation. **Right**, quantification of western blotting analysis with $\log_2$ (fold of change). (n = 3). *$p < 0.05$. **(C) Upper**, representative images of MMP13, ADAMTS4 and COL2A1 labeled immunofluorescence after MAP3K8 knockdown. Scale bars, 200 μm. **Lower**, quantification of immunofluorescence with relative fluorescence intensity (n = 3). *$p < 0.05$. **(D) Upper**, representative images of MMP13, ADAMTS4 and COL2A1 labeled immunofluorescence after the transfection of circRSU1 shRNA or miR-93-5p mimic, with or without overexpressed MAP3K8. **Lower**, quantification of immunofluorescence with relative fluorescence intensity. (n = 3). *$p < 0.05$ compared to negative control (NC) and #$p < 0.05$. Data presented as means ± standard deviation.

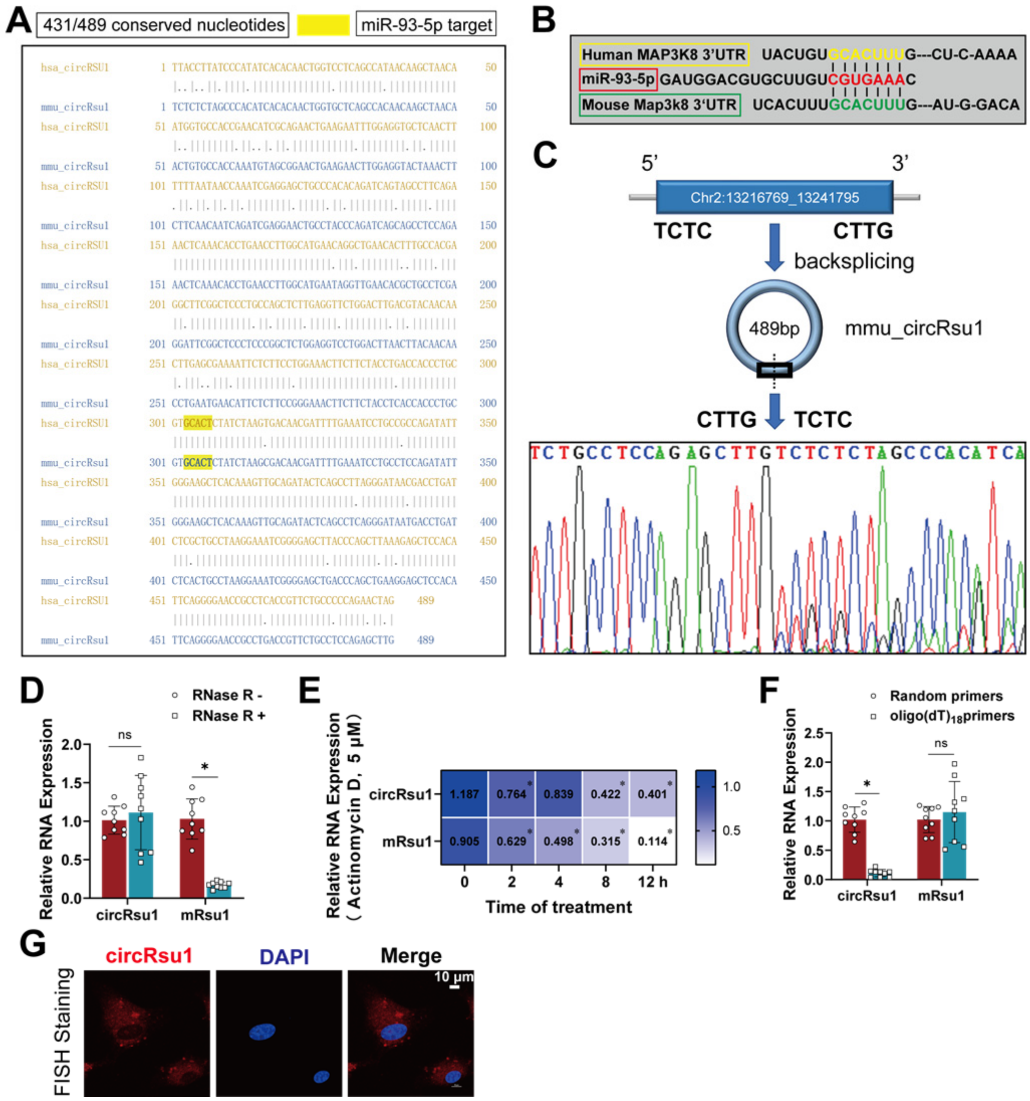

Figure S4.

circRSU1 is conserved in mice. **(A)** Pairwise sequence alignment of hsa_circRSU1 and mmu_circRsu1 using a tool from the EBI website. The sequence highlighted in yellow is targeted by miR-93-5p. **(B)** The conservation of miR-93-5p and its target MAP3K8 gene in humans and mice using the TargetScan database. **(C)** **Upper**, schematic of mmu_circRsu1 backspliced from chromosome 2 in mice as a 489-nucleotide-long circular RNA. **Lower**, backsplicing junction validated by Sanger sequencing. **(D)** Quantitative real-time PCR (qRT-PCR) to confirm the stability of loop structure in circular mmu_circRsu1 compared to linear mmu_mRsu1 after digestion with RNase R. (n = 3). $*p < 0.05$. **(E)** Blue scale heat map showing the stability of mmu_circRsu1 under treatment with actinomycin D (5 μM) for 12 hours. (n = 3). $*p < 0.05$. **(F)** qRT-PCR quantification of mmu_circRsu1 or mmu_mRsu1 relative expression using oligo(dt)$_{18}$ primers or random hexamer primers. (n = 3). $*p < 0.05$. **(G)** Representative images of mmu_circRsu1 fluorescence *in situ* hybridization (FISH) with junction-specific probes in mouse articular chondrocytes. Scale bars, 10 μm. Data presented as means ± standard deviation.

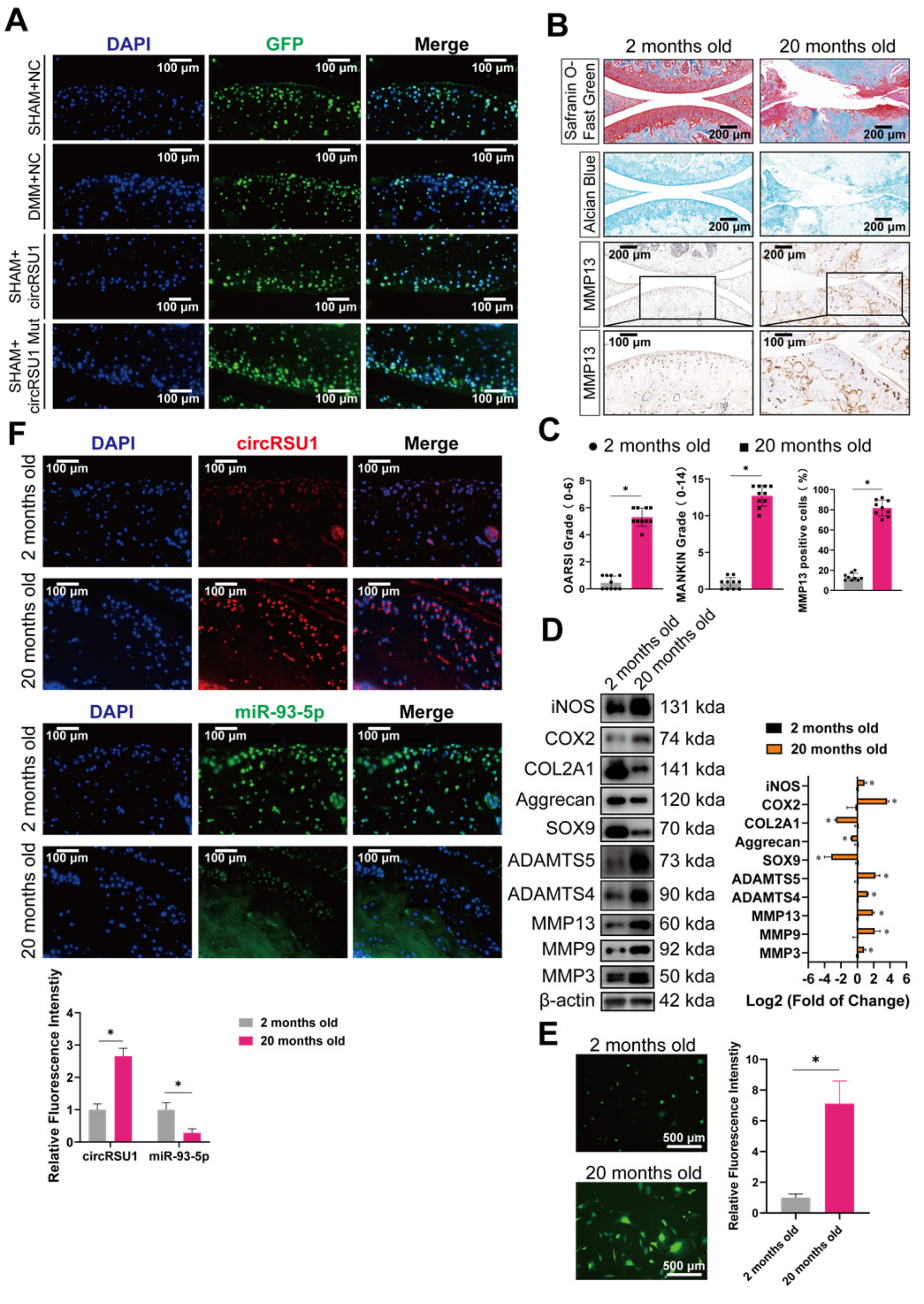

Figure S5.

(A) Representative images of GFP-labeled mouse articular cartilage showing the similar efficiency of infection. Scale bars, 100 μm. **(B)** Representative images of Safranin O/ Fast green staining, Alcian blue staining, and MMP13 labeled knee cartilage from 2-month-old mice or 20-month-old mice. Scale bars, 200 μm. **(C)** OARSI and MANKIN grade used for the assessment

of histological changes. The percentage of MMP13 positive cells used for the quantification of MMP13 labeled cartilage. (n = 10). *p < 0.05. **(D) Left**, western blotting analyses of extracellular matrix (ECM) associated proteins and proinflammatory cytokines. **Right**, quantification of western blotting analyses with $\log_2$ (fold of change). (n = 3). *p < 0.05. **(E) Left**, representative images of reactive oxygen species (ROS) activity detected by DCFH-A probe in mouse chondrocytes. Scale bars, 500 μm. **Right**, quantification of ROS activity with relative fluorescence intensity. (n = 3). *p < 0.05. (**F**) **Upper**, representative images of circRSU1-labeled (red) fluorescence *in situ* hybridization (FISH) staining of mouse articular chondrocytes. **Middle**, representative images of miR-93-5p-labeled (green) FISH staining of mouse articular chondrocytes. Scale bars, 100 μm. **Lower**, quantification of FISH with relative fluorescence intensity. (n = 3). *p < 0.05. Data presented as means ± standard deviation.

Table S1 Antibodies used in the study

Antibody	Catalog Number	Prostitution	Place of Origin
ACTB	60008-1-Ig	Proteintech	Wuhan, China
GAPDH	60004-1-Ig	Proteintech	Wuhan, China
MMP3	ab52915	Abcam	Cambridge, UK
MMP9	10375-2-AP	Proteintech	Wuhan, China
MMP13	ab39012	Abcam	Cambridge, UK
ADAMTS4	ab185722	Abcam	Cambridge, UK
ADAMTS5	ab41037	Abcam	Cambridge, UK
COL2A1	ab34712	Abcam	Cambridge, UK
SOX9	ABE571	Millipore	Massachusetts, USA
Aggrecan	MABT153	Millipore	Massachusetts, USA
MAP3K8	ab137589	Abcam	Cambridge, UK
COX2	12282	CST	Massachusetts, USA
iNOS	ab178945	Abcam	Cambridge, UK
p-NF-κB	3033	CST	Massachusetts, USA
p-ERK1/2	4370	CST	Massachusetts, USA
p-JNK1/2/3	ab124956	Abcam	Cambridge, UK
p-p38	4511	CST	Massachusetts, USA
p-MEK1/2	9154	CST	Massachusetts, USA
p-MSK1	9595	CST	Massachusetts, USA
p-IKKα/β	2697	CST	Massachusetts, USA
p-IκBα	2859	CST	Massachusetts, USA

Table S2 Primers used in the study

Gene	Species	Primer
β-actin-F	human	AGAGCTACGAGCTGCCTGAC
β-actin-R	human	AGCACTGTGTTGGCGTACAG
hsa_circRSU1-F	human	CTGATCTCGCTGCCTAAGGA
hsa_circRSU1-R	human	TGGCACCATTGTTAGCTTGT
hsa_circ_0005354-F	human	ATCATGTGGCTGGACCATCG
hsa_circ_0005354-R	human	GGTCTGCAAAAGCCAACAGG
hsa_circ_0006173-F	human	CCAGACAGGACTTTCTTCTGCT
hsa_circ_0006173-R	human	TGTGGTCAGAATCAGGGCCTA
hsa_circ_0000607-F	human	AGAAATTCAGACTGCAAATGAACA
hsa_circ_0000607-R	human	GCAATCCAGTTTGGGCGTTT
hsa_circ_0002220-F	human	AGGGATTCTGTAGCCCCAAAC
hsa_circ_0002220-R	human	GATCCAGGCCCTCTTTGATGA
hsa_circ_0006646-F	human	TTCACGTGGATATGGGCGTC
hsa_circ_0006646-R	human	AGTTGGGGTCAAGGTAAGCAG
hsa_circ_0008702-F	human	AGAATCCAAATGCGCACGAG
hsa_circ_0008702-R	human	GAAGGGCTTCTGGTCTGGTC
hsa_circ_0002431-F	human	AAGGTAGCAGTGACACCTCC
hsa_circ_0002431-R	human	TTGACTTGCAATCCTCAGAGAGT
hsa_circ_0001472-F	human	TGAGCGAATAGAGAGAGAATCAGC
hsa_circ_0001472-R	human	TCAGGTGCCAGCTGTCATTA
hsa_circ_0002359-F	human	TGTTGCTGCTGATGAAGACGTT
hsa_circ_0002359-R	human	GATCAGGGTCTGGGCGAACTA
hsa_circ_0003391-F	human	ACACAACATCTTCCCCCACA
hsa_circ_0003391-R	human	GAAGGGGTCACCGACATGG
hsa_circ_0002664-F	human	ACTATGTTATTCGAGGTTGAGCA
hsa_circ_0002664-R	human	TGAGAGGGTAGCCCTTAGCA
hsa_circ_0003715-F	human	GCCTGGAGTAGGAGTTTAAGGA
hsa_circ_0003715-R	human	TCCAGCAAATATCAACACAAGTTGG
hsa_circ_0004182-F	human	CCTGCAGCAATCCCTTTGAG
hsa_circ_0004182-R	human	TAGAGAAATGCTGGGCCCTTG
hsa_circ_0001658-F	human	CATCTCCCTCTCCTGTTGGC
hsa_circ_0001658-R	human	CCACCTAGGAGGAACTGACAA
hsa_circ_0009140-F	human	AGGGGAAGATGAAGGAGCCAT
hsa_circ_0009140-R	human	GCTGGAGAGTTTTCCACTCCT
hsa_circ_0001742-F	human	GTGGGATTTGTTTTGTGGGCT
hsa_circ_0001742-R	human	TCTTGGGGTTGTCTGTCCGA
hsa_circ_0001614-F	human	TCATACAGTGCACACTGGTTTTT
hsa_circ_0001614-R	human	TCTGTATGACAGCATTTTCATGGT
hsa_circ_0001788-F	human	GCTAGAAAAAGCATCAAATCCCA
hsa_circ_0001788-R	human	GGCTGGGAGATCCCATCAATTT
hsa_circ_0000257-F	human	GGAGCAGACCAAGGCAGCG
hsa_circ_0000257-R	human	CGTCAAAGATCACGACTGTCCC

Gene	Species	Primer
hsa_circ_0008539-F	human	ATGCCGACTCTCATACAGGC
hsa_circ_0008539-R	human	TTGATCCGCAAGAGATGCCC
hsa_circ_0001861-F	human	GATCCGAGTTGGCTACACCC
hsa_circ_0001861-R	human	CTCCACCTCACAGTTCTTCACT
hsa_circ_0000836-F	human	AAGCTGGAGCAGATGTTACC
hsa_circ_0000836-R	human	GCACACTTCCATCGAATGCC
hsa_circ_0007312-F	human	CAAGATTAGCACAGATTTGCTTGTA
hsa_circ_0007312-R	human	TTGCAGACACTTTAGGCACTGA
hsa_circ_0001447-F	human	AAAAGAACTGCTGCCCAGTGA
hsa_circ_0001447-R	human	ATCGAGACATGGGAGTCCTG
hsa_circ_0001495-F	human	TGGTGAATGGAATAATTGTGTGCC
hsa_circ_0001495-R	human	AGTCACCAATTTCTGGAGGGT
MMP3-F	human	CCTACAAGGAGGCAGGCAAG
MMP3-R	human	CCCGTCACCTCCAATCCAAG
MMP9-F	human	GTACTCGACCTGTACCAGCG
MMP9-R	human	GTACTCGACCTGTACCAGCG
MMP13-F	human	TCGGCCACTCCTTAGGTCTT
MMP13-R	human	AAGTGGCTTTTGCCGGTGTA
ADAMTS4-F	human	AACGTCAAGGCTCCTCTTGG
ADAMTS4-R	human	TGACAGGATTGCGGATGCTT
ADAMTS5-F	human	CCGGAGCCACTGCTTCTATC
ADAMTS5-R	human	ACCCCCACAGAGGTCAAAGA
COL2A1-F	human	CCAGATGACCTTCCTACGCC
COL2A1-R	human	TTCAGGGCAGTGTACGTGAAC
SOX9-F	human	GCTCTGGAGACTTCTGAACGA
SOX9-R	human	CCGTTCTTCACCGACTTCCT
Aggrecan-F	human	AAGGGCGAGTGGAATGATGT
Aggrecan-R	human	CGTTTGTAGGTGGTGGCTGTG
IL-1β-F	human	TCGCCAGTGAAATGATGGCT
IL-1β-R	human	TGGAAGGAGCACTTCATCTGTT
TNFα-F	human	TAGCCCATGTTGTAGCAAACC
TNFα-R	human	GCTCTTGATGGCAGAGAGGA
IL6-F	human	TAGTGAGGAACAAGCCAGAGC
IL6-R	human	TATTGCATCTAGATTCTTTGCCTTT
mRSU1-F	human	GCTGAACACTTTGCCACGAG
mRSU1-R	human	AGCTTCCCAATATCTGGCGG
miR-93-5p-F	human	CAAAGTGCTGTTCGTGCAGGTAG
miR-433-5p-F	human	CGTACGGTGAGCCTGTCATTATTC
miR-449c-5p-F	human	TAGGCAGTGTATTGCTAGCGG
miR-637-F	human	TATACTGGGGGCTTTCGGG
miR-1207-5p-F	human	TATATATGGCAGGGAGGCTGGG
miR-4763-3p-F	human	TATAAGGCAGGGGCTGGTG
hsa-U6-F	human	AGAGAAGATTAGCATGGCCCCT

Gene	Species	Primer
AKT1-F	human	AGGAGGTTTTTGGGCTTGCG
AKT1-R	human	GATGTACTCCCCTCGTTTGTGC
AKT2-F	human	TGCCGGTGACAGGTGAATAC
AKT2-R	human	AGGCAGCGTATGACAAAGGT
AKT3-F	human	TGGATGCCTCTACAACCCATC
AKT3-R	human	GTGTGTGCCACTTCATCCTTTG
CREB1-F	human	ACCAAGTTGTTGTTCAAGGTACT
CREB1-R	human	TGTTACCATCTTCAAACTGACGTT
ERK1-F	human	CTCCAAGGGCTATACCAAGTC
ERK1-R	human	CTTAGGTAGGTCATCCAGCTCC
ERK2-F	human	ATTCCAAGGGCTACACCAAGT
ERK2-R	human	GATGTCTGAGCACGTCCAGT
GSK3A-F	human	CAGTGGCGAGAAGAAAGACGA
GSK3A-R	human	CTGCGGAAGAGCTGGTACAT
GSK3B-F	human	GGTCCGAGGAGAACCCAATG
GSK3B-R	human	GGTCGGAAGACCTTAGTCCAA
HSP27-F	human	ACGCGGAAATACACGCTGC
HSP27-R	human	TTACTTGGCGGCAGTCTCAT
JNK1-F	human	TCTCCTTTAGGTGCAGCAGTG
JNK1-R	human	TAACCGACTCCCCATCCCTC
JNK2-F	human	CCCTTCGGGATATTGCAGGA
JNK2-R	human	AATGCAGCACAAACAATCCC
JNK3-F	human	ATGGTACAGGCAGCATCACG
JNK3-R	human	CACACTAGCCATTCCGTGCC
MKK3-F	human	CTAGATTAGTCTCCACCGCCG
MKK3-R	human	GACCAGCCTCTCTTTGGACT
MKK6-F	human	GTTTCTCCTTGCCGAAGTGTG
MKK6-R	human	ATCTCGAGGTGGTGTGGAACT
MSK2-F	human	CTTCTGTGGCACCATCGAGT
MSK2-R	human	AAGGGCGAGTCCTGCATCAT
1p38α-F	human	TTAAGACTCGTTGGAACCCCAG
1p38α-R	human	TAGGTCAGGCTTTTCCACTCATC
2p38β-F	human	CCTTCCAGTCGCTGATCCAC
2p38β-R	human	CCAGAAGCCCGATGACGTTC
3p38γ-F	human	TGAGGTATATCCACGCTGCC
3p38γ-R	human	AGTTCTTGGCCTCATCGCTC
4p38δ-F	human	ATCAAGAAGCTGAGCCGACC
4p38δ-R	human	GCCCAATGACGTTCTCATGC
p53-F	human	CTGAGGACATGGCAGGAGTG
p53-R	human	ACAATGTTCCATGCCAAGTTCA
RPS6KB1-F	human	CTGAGGACATGGCAGGAGTG

Gene	Species	Primer
RPS6KB1-R	human	ACAATGTTCCATGCCAAGTTCA
RPS6KA1-F	human	CTCATGGAGCTAGTGCCTCT
RPS6KA1-R	human	TTTGCTCAGGCCAAAGTCAGT
RPS6KA3-F	human	CGCACAAGGGGTGGTTCATA
RPS6KA3-R	human	TTCCGCTACCTATTCGTGCC
MTOR-F	human	CCATCCGTGTGTTAGGGCTT
MTOR-R	human	CTAGCGCTGCCTTTCGAGAT
NF-κB-F	human	GGAAGAGGAGGTTTCGCCAC
NF-κB-R	human	GCCCCTTATACACGCCTCTG
circRsu1-F	mouse	ACCCTGCGTGCACTCTATCT
circRsu1-R	mouse	CCAGTTGTGTGATGTGGGCTA
mRsu1-F	mouse	GGTCCCTAATCACACCCACC
mRsu1-R	mouse	GGCTAGAGAGAACAAGCCGTT

Table S3A General Conditions of Patients

NO.	Gender	Age	BMI	WOMAC GRADE （100 points total）			Outerbridge GRADE		Kellgren & Lawrence Grade	
				Pain	Stiffness	Activity	Medial	Lateral	Medial	Lateral
1	M	64	25.7	10	4	35	3	1	3	1
2	M	61	24.1	11	3	30	3	1	3	1
3	M	69	26.9	13	4	32	3	1	3	1
4	F	63	27.4	9	4	30	4	2	4	2
5	F	65	25.3	10	5	38	3	1	3	1
6	F	66	27.1	10	3	36	3	1	3	2
7	M	60	28.4	12	5	30	3	1	3	1
8	F	67	29.2	13	3	32	3	1	3	1
9	M	60	23.1	12	2	31	3	1	3	1
10	F	67	23.7	11	2	40	3	1	3	1
11	M	62	26.3	10	2	33	3	1	3	1
12	F	63	25.8	12	3	32	3	1	3	1
13	M	67	27.6	13	2	37	3	1	3	1
14	M	60	22.9	9	3	37	3	1	3	1
15	F	67	26.4	14	5	39	4	2	4	2
16	F	73	25.3	18	6	45	3	2	3	2
17	F	83	24.8	17	7	55	4	2	4	2
18	F	77	23.9	19	8	60	3	2	3	2
19	M	81	22.6	18	7	58	4	3	4	3
20	F	81	24.3	18	7	59	4	3	4	3
21	M	77	29.4	17	8	52	3	2	3	2
22	M	82	28.9	20	8	55	4	2	4	2
23	M	73	29.8	18	5	48	3	2	3	2

NO.	Gender	Age	BMI	WOMAC GRADE (100 points total)			Outerbridge GRADE		Kellgren & Lawrence Grade	
				Pain	Stiffness	Activity	Medial	Lateral	Medial	Lateral
24	F	76	24.3	17	6	58	3	2	4	2
25	M	72	25.7	15	6	50	3	2	4	2
26	F	72	25.7	15	5	56	3	2	4	2
27	F	85	28.3	18	6	62	4	3	4	3
28	M	85	23.1	20	7	63	4	3	4	3
29	M	75	23.4	20	8	56	4	2	4	3
30	F	72	22.1	17	7	51	3	2	4	2

Table S3B Summary of General Conditions of Patients

Item	Younger Patients (60-69 years old)	Older Patients (70-85 years old)
Gender	8/7 (Male/Femal)	7/8 (Male/Femal)
Age	64.07 ± 2.93	77.60 ± 4.67 *
BMI	25.99 ± 1.84	25.44 ± 2.44
